# Enhancing CAR-T cell metabolism to overcome hypoxic conditions in the brain tumor microenvironment

**DOI:** 10.1172/jci.insight.177141

**Published:** 2024-02-22

**Authors:** Ryusuke Hatae, Keith Kyewalabye, Akane Yamamichi, Tiffany Chen, Su Phyu, Pavlina Chuntova, Takahide Nejo, Lauren S. Levine, Matthew H. Spitzer, Hideho Okada

**Affiliations:** 1 Department of Neurological Surgery,; 2 Department of Otolaryngology-Head and Neck Surgery, and; 3 Department of Microbiology and Immunology, UCSF, San Francisco, California, USA.; 4 The Parker Institute for Cancer Immunotherapy, San Francisco, California, USA.

**Keywords:** Immunology, Oncology, Brain cancer, Cancer immunotherapy, Hypoxia

## Abstract

The efficacy of chimeric antigen receptor T cell (CAR-T) therapy has been limited against brain tumors to date. CAR-T cells infiltrating syngeneic intracerebral SB28 EGFRvIII gliomas revealed impaired mitochondrial ATP production and a markedly hypoxic status compared with ones migrating to subcutaneous tumors. Drug screenings to improve metabolic states of T cells under hypoxic conditions led us to evaluate the combination of the AMPK activator metformin and the mTOR inhibitor rapamycin (Met+Rap). Met+Rap–pretreated mouse CAR-T cells showed activated PPAR-γ coactivator 1α (PGC-1α) through mTOR inhibition and AMPK activation, and a higher level of mitochondrial spare respiratory capacity than those pretreated with individual drugs or without pretreatment. Moreover, Met+Rap–pretreated CAR-T cells demonstrated persistent and effective antiglioma cytotoxic activities in the hypoxic condition. Furthermore, a single intravenous infusion of Met+Rap–pretreated CAR-T cells significantly extended the survival of mice bearing intracerebral SB28 EGFRvIII gliomas. Mass cytometric analyses highlighted increased glioma-infiltrating CAR-T cells in the Met+Rap group, with fewer Ly6c^+^CD11b^+^ monocytic myeloid-derived suppressor cells in the tumors. Finally, human CAR-T cells pretreated with Met+Rap recapitulated the observations with murine CAR-T cells, demonstrating improved functions under in vitro hypoxic conditions. These findings advocate for translational and clinical exploration of Met+Rap–pretreated CAR-T cells in human trials.

## Abstract 2

Pretreatment of in SB28-EGFRvIII glioma mice with a combination of metformin and rapamycin boosts mitochondrial ATP in CAR-T cells, thus improving efficacy and survival.

## Introduction

Glioblastoma multiforme (GBM) is an aggressive and lethal brain tumor despite the combined standard of care with maximum surgical resection, radiation, and chemotherapy. One potential approach to treating GBM is immunotherapy; however, despite promising results in several other types of cancer, immunotherapy has not been effective against GBM (1). One of the key challenges to successful immunotherapy in GBM is the highly immunosuppressive tumor microenvironment characterized by a number of mechanisms (2), including hypoxic conditions (3). As such, recent research has focused on developing innovative strategies to overcome these challenges and improve the effectiveness of immunotherapy (4, 5).

Chimeric antigen receptor T cell (CAR-T) therapy has demonstrated significant efficacy against hematologic malignancies (6). However, its therapeutic potential remains limited against solid tumors, including brain neoplasms (5). The metabolic status of immune cells has recently been recognized as a critical factor in cancer immunotherapy. Glycolytic metabolism is essential for effector T cells, and oxidative phosphorylation (OXPHOS), which occurs in mitochondria, is crucial for the high survival capability of memory T cells (7). Furthermore, glycolysis and OXPHOS are known to be decreased in exhausted T cells (8). In the tumor microenvironment, hypoxic conditions and chronic antigen stimulation rapidly diminish T cell mitochondrial function and lead to exhaustion (9). Therefore, we hypothesized that enhancing the mitochondrial function of CAR-T cells could prevent them from becoming exhausted in the hypoxic microenvironment of GBM. To address this hypothesis, in this study, we investigated the preconditioning of CAR-T cells with metabolic regulators before infusion and examined its translational potential.

## Results

### CAR-T cells lose OXPHOS activity in the glioma microenvironment.

**Figure 1:**
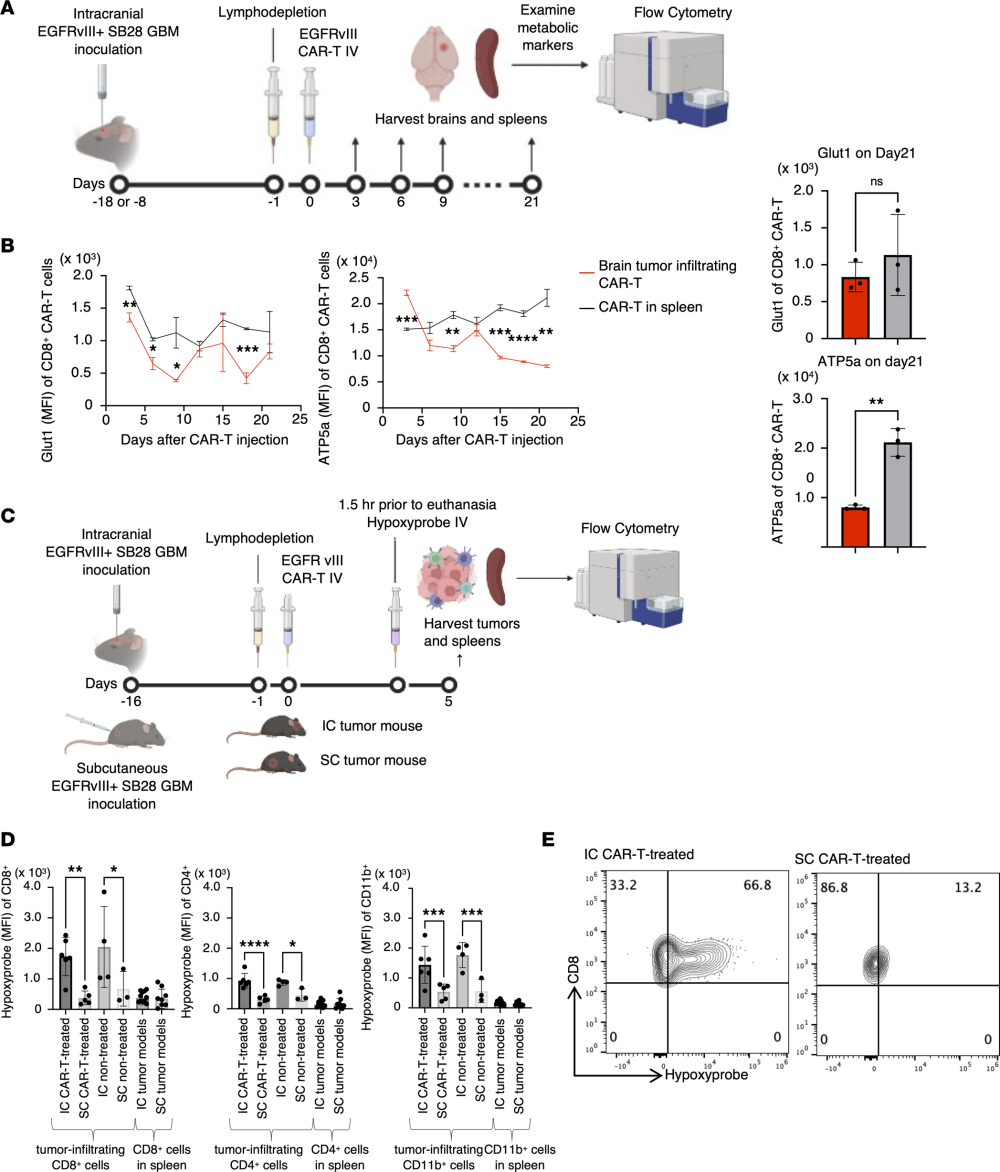
Exhaustion of CAR-T cells is associated with reduced OXPHOS activity in the hypoxic glioma microenvironment. (**A**) The experimental design to evaluate glioma-infiltrating CAR-T cells. IV, intravenous. (**B**) Longitudinal changes in Glut1 and ATP5a, markers of the glycolytic system and OXPHOS, respectively, in glioma-infiltrating CD8^+^ CAR-T cells (left panels). Expression of Glut1 (top right) and ATP5a (bottom right) by mean fluorescence intensity (MFI) in CD8^+^ CAR-T cells extracted from the spleen (gray) and tumor (red) on day 21. (**C**) The design for analyzing hypoxic conditions in vivo. (**D**) Uptake of hypoxyprobe by CD8^+^ (left), CD4^+^ (middle), or CD11b^+^ (right) leukocytes infiltrating intracranial tumor model (IC) or subcutaneous tumor model (SC) SB28 mEGFRvIII gliomas or spleens of glioma-bearing mice. Data are presented as mean ± SD. *P < 0.05; **P < 0.01; ***P < 0.001, ****P < 0.0001 by unpaired, 2-tailed *t* test (**B**) or 1-way ANOVA followed by Tukey’s multiple comparison test (**D**). (**E**) Representative histograms (IC or SC tumors in CAR-treated mice) showing the positive staining with hypoxyprobe on CD8^+^ BILs but not on CD8^+^ CAR-T cells isolated from SC tumors.

To investigate the metabolic status within the tumor microenvironment, we leveraged the experimental system of anti-EGFRvIII CAR-T cells isolated from EGFRvIII CAR-T–transgenic mice that we established previously (Supplemental Figure 1A; supplemental material available online with this article; https://doi.org/10.1172/jci.insight.177141DS1) (10). This system allows us to obtain a large number of CAR-T cells with consistent quality, thus enabling us to perform extensive experiments with high scientific rigor. We evaluated the metabolic status of CAR-T cells following intravenous (i.v.) infusion into syngeneic C57BL/6J mice bearing intracerebral SB28 tumors expressing human epidermal growth factor variant III (SB28 hEGFRvIII). The mice received a single i.v. infusion of 3 × 10^6^ anti-EGFRvIII CAR-T cells. A cohort of 3 mice was sacrificed every 3 days after infusion up to 21 days, the glioma tissues were harvested, and the metabolic status of brain tumor–infiltrating CAR-T cells was evaluated by flow cytometry (Figure 1A). The expression levels of the glycolytic marker glucose transporter 1 (Glut1) were not significantly different between the CD8^+^ CAR-T cells isolated from the glioma tissues and those derived from the spleen (Figure 1B). On the other hand, the expression levels of ATP synthase (ATP5a), a marker of OXPHOS, in the glioma-infiltrating CD8^+^ CAR-T cells continuously decreased over time, while those in the spleen-derived CD8^+^ CAR-T cells did not show such decreasing trends (Figure 1B). Moreover, CD4^+^ CAR-T cells demonstrated similar findings (Supplemental Figure 1B).

### The brain tumor microenvironment is hypoxic.

Since OXPHOS is a cellular process that produces a large amount of ATP by utilizing oxygen in the intracellular mitochondria, we hypothesized that the decreased OXPHOS activity in CAR-T cells might be due to the hypoxic glioma microenvironment. To address this hypothesis, in mice bearing day 16 SB28 mEGFRvIII tumors in the brain or subcutaneous space in the right flank of syngeneic C57BL/6J mice, we i.v. administered anti-EGFRvIII CAR-T cells. We used SB28 mEGFRvIII in our in vivo studies because of the more consistent luciferase expression in the mEGFRvIII version than SB28 hEGFRvIII (data not shown). Five days later, hypoxyprobe, an agent specifically taken up by hypoxic cells, was administered to the mice, followed by harvesting of the glioma tissues and spleens at 1.5 hours after the hypoxyprobe infusion (Figure 1C). Flow cytometric evaluations of CD8^+^, CD4^+^, and CD11b^+^ cells isolated from these organs revealed that CD8^+^, CD4^+^, and CD11b^+^ populations in the intracerebral glioma tissue were significantly more hypoxic than those in subcutaneous tumors regardless of CAR-T cell administration (Figure 1, D and E).

### Pretreatment of CAR-T cells with metformin and rapamycin improves the sustained function of CAR-T cells in hypoxic conditions.

**Figure 2:**
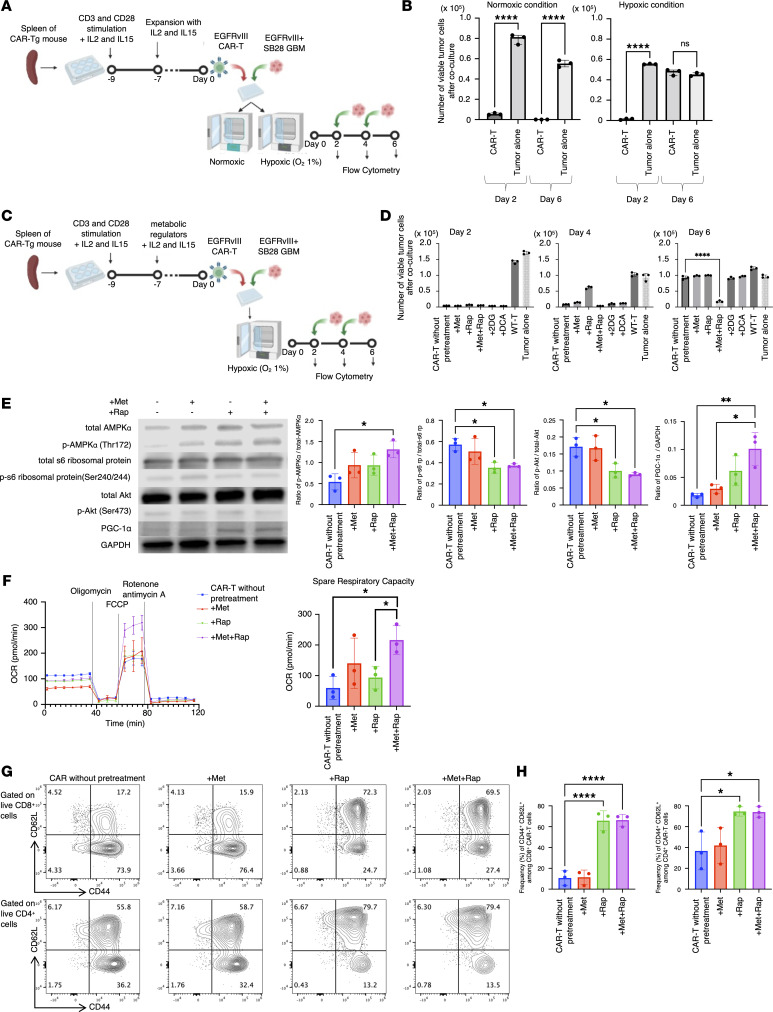
A combination of metformin and rapamycin (Met+Rap) promotes the persistent function of CAR-T cells in hypoxic condition. (**A**) The design of the coculture experiment. (**B**) The number of viable tumor cells after coculture under normoxic condition (left) or hypoxic condition (right). Data are presented as mean ± SD. (**C**) The design for evaluating metabolic regulators. (**D**) The number of viable tumor cells on day 2 (left), day 4 (middle), and day 6 (right). Data are presented as mean ± SD. (**E**) Representative immunoblot of phosphorylation of AMPKα, S6 ribosomal protein, and Akt, and the expression of total AMPKα, total S6 ribosomal protein, total Akt, and PGC-1α in pretreated CD8^+^ CAR-T cells. The bar charts represent quantitative comparisons between the groups (*n* = 3/group). For Akt, blots were run on parallel gels using the same samples. Data are presented as mean ± SD. (**F**) Left: OCR of CAR-T cells was measured by Seahorse XFe96 analyzer on day 0. Data presented as mean ± SEM. Right: Spare respiratory capacity levels were calculated. Data are presented as mean ± SD. (**G**) Flow cytometric plots for CD44 and CD62L in CD8^+^ and CD4^+^ CAR-T cells on day 0. (**H**) The bar graphs illustrate the comparative frequencies of the CD44^+^CD62L^+^ cell population across the groups (*n* = 3/group). Data are presented as mean ± SD. *P < 0.05; **P < 0.01; ****P < 0.0001 by 1-way ANOVA followed by Tukey’s multiple-comparison test (**B**, **D**, **E**, and **H**) or unpaired, 2-tailed *t* test (**F**).

We then hypothesized that treating CAR-T cells with mitochondria-activating drugs before infusion ex vivo would improve the function of CAR-T cells in hypoxic conditions. In vitro, under the normoxic condition (Figure 2A), CAR-T cells maintained their potent cytotoxic function against the glioma cells, even after new glioma cells were introduced every 2 days for 3 cycles. On the other hand, under hypoxic conditions, CAR-T cells lost their antitumor effect after 3 cycles of tumor cell challenge (Figure 2B). To develop a strategy to overcome reduced cytotoxicity in hypoxic conditions, we selected and tested metformin (Met), rapamycin (Rap), 2-deoxyglucose (2DG), and dichloroacetate (DCA) because these have been studied in combination with inhibitory anti–PD-1 antibodies and are expected to activate mitochondria (Table 1) (11). We treated CAR-T cells with each of these OXPHOS-activating metabolic regulators prior to coculturing them with SB28 mEGFRvIII tumor cells to evaluate whether the antitumor effect could improve under the hypoxic condition (Figure 2C). Among the drugs evaluated, only the combination of Met and Rap (Met+Rap) enhanced the persistent antitumor effect of CAR-T cells under the hypoxic condition (Figure 2D).

### Ex vivo Met+Rap pretreatment enhances the mitochondrial respiratory capacity by activating AMPK and PGC-1α in CAR-T cells.

To unravel the mechanism underlying the effect of Met+Rap on CAR-T cells, we investigated the activation of AMPK and mTOR pathways in CD8^+^ CAR-T cells after treatment with Met or Rap alone, or the combination of Met+Rap. The Met+Rap treatment activated AMPKα and inhibited phosphorylated S6 (p-S6) ribosomal protein and p-Akt Ser473 (downstream targets of mammalian target of rapamycin complex 1 [mTORC1] and mTORC2, respectively) (Figure 2E). Furthermore, Met+Rap pretreatment led to the upregulation of PPAR-γ coactivator 1α (PGC-1α), the master regulator of mitochondrial biogenesis and function (12), likely due to the combined effects of Met-induced AMPK activation and Rap-induced mTOR inhibition. It has been reported that T cells overexpressing PGC-1α are less prone to exhaustion in the tumor microenvironment (13). Therefore, based on the upregulation of PGC-1α, we hypothesized that Met+Rap combination treatment would improve the mitochondrial energy metabolism of CAR-T cells, leading to better persistence and antitumor cytotoxicity even under hypoxic conditions. To address this, we conducted the Seahorse assay–based Mito Stress Test with CAR-T cells pretreated with metabolic regulators and found that Met+Rap treatment significantly increased the spare respiratory capacity (SRC) of CAR-T cells (Figure 2F). This observation was encouraging, because previous studies have shown that cells with a higher SRC can survive better under hypoxic conditions (14). Importantly, these findings were consistently observed in CAR-T cells isolated from both male and female mice (Supplemental Figure 2, A–C).

Several publications reported the ability of Met or Rap to enhance the central memory T cell population (15, 16). Consistent with these findings, our data revealed that CAR-T cells pretreated with Rap or Met+Rap demonstrated a significantly higher percentage of CD44^+^CD62L^+^ central memory T cells than nontreated CAR-T cells (Figure 2, G and H). In contrast, those pretreated with Met alone failed to show an increase in this population. Memory T cells generally exhibit a higher mitochondrial SRC (17). While the Rap and Met+Rap pretreatments gave rise to similar numbers for the central memory population, the Met+Rap pretreatment augmented the SRC relative to Rap alone, suggesting a more pronounced mitochondrial activation induced by the combined Met+Rap pretreatment (Figure 2F and Supplemental Figure 2C). Crucially, it was ascertained that treatment of CAR-T cells with Met, Rap, or Met+Rap did not markedly affect cell proliferation during the expansion phase of mouse CAR-T cells (Supplemental Figure 3).

### Metabolic regulator therapy promotes the resistance of CAR-T cells against exhaustion.

To investigate whether Met+Rap treatment would save CAR-T cells from exhaustion, we first treated CAR-T cells with Met or Rap alone, Met+Rap, or without any of these for 1 week during the expansion, maintained them in a drug-free condition for the next 6 days, and profiled them by flow cytometry (Supplemental Figure 4A). CAR-T cells without pretreatment showed more effector memory type cells and higher levels of Tim3, PD-1, IFN-γ, and TNF-α than WT (non-CAR) T cells (Supplemental Figure 4B). These observations are likely attributable to nonspecific activation signals triggered by the CAR, such as tonic signaling (18), which was mitigated by Rap and Met+Rap treatment (Supplemental Figure 4B). Moreover, while the number of untreated CAR-T cells significantly decreased, nearly 50% over a 6-day hypoxic culture period, the number of Met+Rap–pretreated CAR-T cells nearly doubled under the identical hypoxic condition (Supplemental Figure 4C). This observation underscores the potential of Met+Rap pretreatment to promote CAR-T cell proliferation, even under hypoxic conditions.

**Figure 3:**
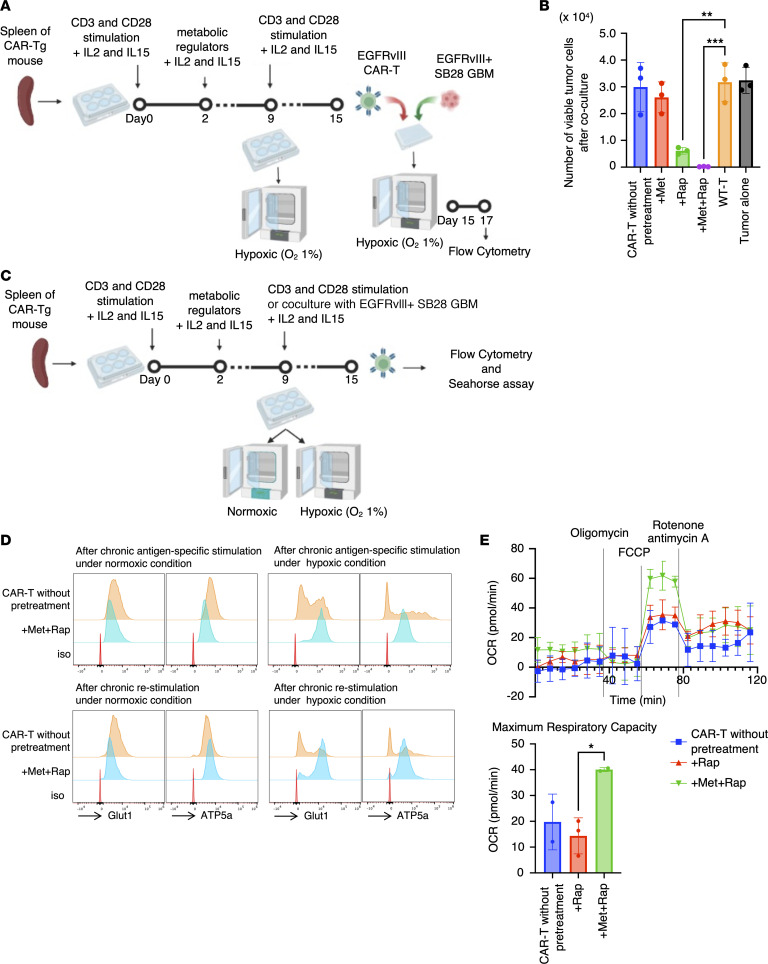
Chronic hypoxic condition–induced exhaustion of CAR-T cells is mitigated by metabolic regulators. (**A**) Coculture experimental design following chronic restimulation with anti-CD3/anti-CD28 Dynabeads under hypoxic condition. (**B**) Graph showing the number of live tumor cells after coculture. Data are presented as mean ± SD. (**C**) Experimental design. (**D**) Flow cytometric data for metabolic markers Glut1 and ATP5a in CD8^+^ CAR-T cells with and without pretreatment with Met+Rap on day 15 with chronic antigen-specific stimulation with SB28 mEGFRvIII gliomas (top) and CD3/CD28 stimulation (bottom) in normoxic (left) and hypoxic conditions (right). (**E**) Top: OCR of day 15 CAR-T cells after the chronic restimulation under the hypoxic condition was measured by Seahorse XFe96 analyzer. Data are presented as mean ± SEM. Bottom: Maximum respiratory capacity was calculated. Data are presented as mean ± SD. *P < 0.05; **P < 0.01; ***P < 0.001 by 1-way ANOVA followed by Tukey’s multiple-comparison test (**B**) or unpaired, 2-tailed *t* test (**E**).

Next, to investigate their exhaustion resistance, we tested chronic stimulation of CAR-T cells pretreated with the metabolic regulators. After pretreatment with the drugs, to induce exhaustion, CAR-T cells were chronically restimulated with anti-CD3/anti-CD28 antibody–conjugated beads under the hypoxic condition for 6 days following the expansion (9) (Figure 3A). Subsequently, their cytotoxic activity was evaluated against SB28 mEGFRvIII cells under hypoxia. CAR-T cells without pretreatment revealed the lowest level of cytolytic capability, suggesting their exhaustion status. Conversely, those pretreated with Rap and Met+Rap retained their antitumor effect even after chronic CD3/CD28 stimulation, indicating their better tolerance against exhaustion (Figure 3B).

Previous studies have reported that exhausted T cells exhibit suppressed mitochondrial respiration and glycolysis (9, 19). To investigate whether the metabolic regulators prevent decreases in OXPHOS, we chronically stimulated CAR-T cells with SB28 mEGFRvIII gliomas or anti-CD3/anti-CD28 beads under hypoxia (Figure 3C). Under normoxia, there was no remarkable difference in the levels of Glut1 and ATP5a between untreated and Met+Rap–pretreated CAR-T cells (Figure 3D). However, under hypoxia, chronic antigen-specific or CD3/CD28 stimulation led to reduced Glut1 and ATP5a in untreated CAR-T cells, suggesting an exhausted status. In contrast, Met+Rap–pretreated CAR-T cells maintained high levels of Glut1 and ATP5a even after antigen-specific or CD3/CD28 stimulation (Figure 3D). Moreover, the Seahorse assay–based Mito Stress Test demonstrated that the enhanced maximum respiratory capacity after Met+Rap treatment, but not Rap treatment, persisted after the 6-day course of chronic hypoxic CD3/CD28 stimulation (Figure 3E). These data collectively suggest that the potent resistance to exhaustion in CAR-T cells is induced through the improved metabolic status due to the treatment with Met+Rap.

### In vitro treatment of CAR-T cells with Met+Rap improves the survival of mice bearing intracerebral glioma following i.v. infusion.

**Figure 4:**
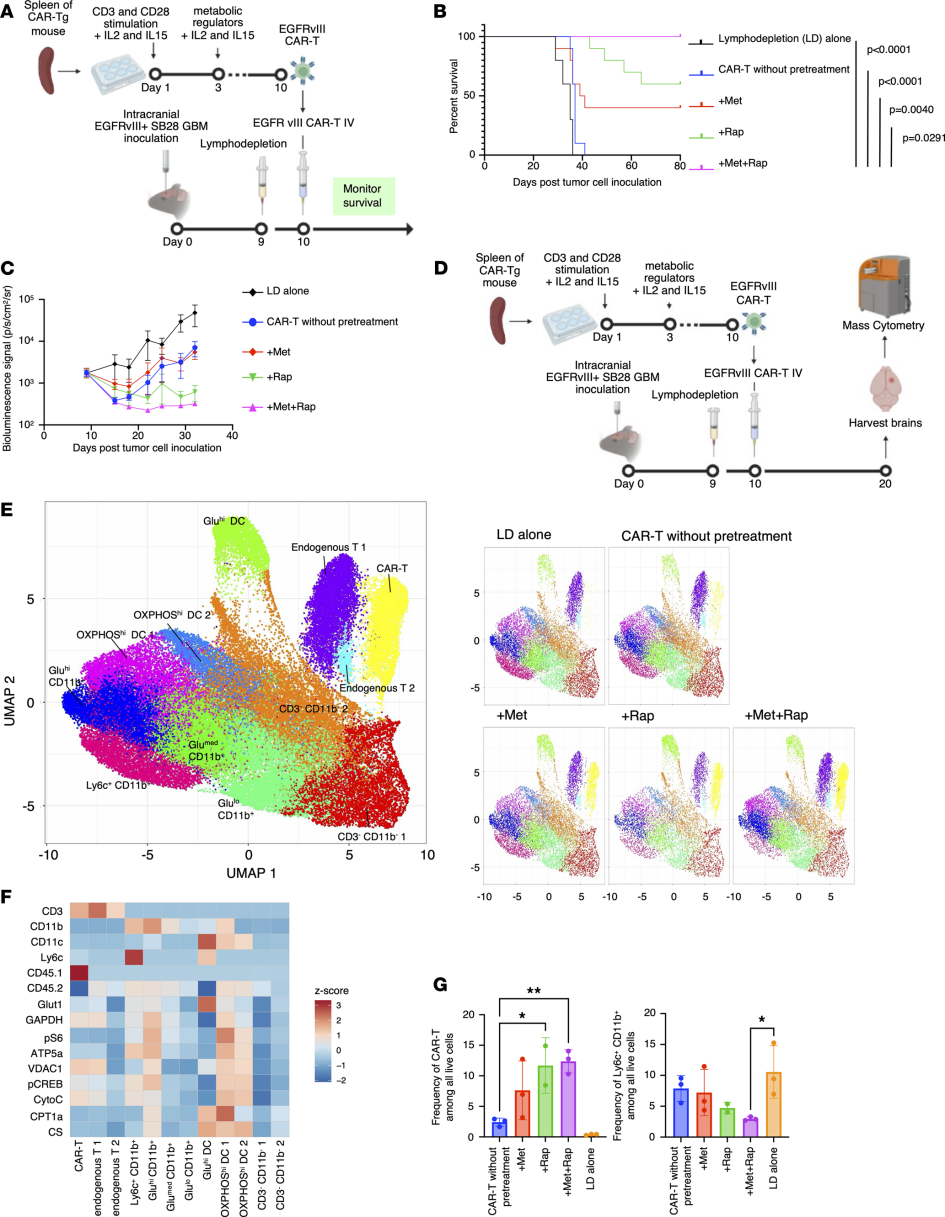
Pretreatment of CAR-T cells with Met+Rap extended the survival of glioma-bearing mice and enhanced their glioma infiltration. (**A**) Schematic of the treatment protocol for the survival study with the SB28 mEGFRvIII (murine EGFRvIII) glioma. IV, intravenous. (**B**) Kaplan-Meier curves: lymphodepletion (LD) group (median survival [MS] = 35 days, *n* = 10), CAR-T cells without pretreatment (MS = 37 days, *n* = 10), with Met-pretreated CAR-T (MS = 40 days, *n* = 10), with Rap-pretreated CAR-T (MS = not reached, *n* = 10), and with Met+Rap-pretreated CAR-T (MS = not reached, *n* = 10). (**C**) Tumor size was measured by luciferase bioluminescence imaging over time as the number of photons per second per square centimeter per steradian (p/s/cm^2^/sr). Data presented as mean ± SEM. (**D**) The design of the mass cytometric analysis. (**E**) Uniform manifold approximation and projection (UMAP) plot of BILs. The UMAP in the left penal shows all samples combined, while the panels on the right side show each treatment group separately. (**F**) Heatmap visualizing the relative expression (*z* score) of immune cell markers and metabolic markers in each subpopulation. Each cluster was annotated based on the expression status of the markers as indicated in the left panel. Clusters with similar marker expression levels are indicated with labels 1 and 2. (**G**) Frequencies of SB28 mEGFRvIII–infiltrating CAR-T cells (left panel) and Ly6c^+^ CD11b^+^ monocytic myeloid-derived suppressor cells (MDSCs; right panel) among the pretreatment types. Data are presented as mean ± SD. *P < 0.05; **P < 0.01 by 1-way ANOVA followed by Tukey’s multiple-comparison test.

To determine whether the Met+Rap treatment of CAR-T cells improves their functions in vivo, C57BL/6J mice bearing intracerebral SB28 mEGFRvIII gliomas received a single i.v. infusion of 1 × 10^6^ CAR-T cells pretreated with Met or Rap alone, Met+Rap, or without any of these (Figure 4A). As shown in Figure 4B, the infusion with Met+Rap–pretreated CAR-T cells resulted in all mice surviving 80 days after tumor inoculation (median survival not reached), while all the mice receiving untreated CAR-T cells died by 41 days after tumor implantation (median survival 37 days) (log-rank, *P* < 0.0001).

Previous reports have indicated that pretreatment with IL-15 or Rap preserves the stem cell memory phenotype and improves therapeutic outcomes in vivo (20). However, the Met+Rap treatment group demonstrated significantly more extended survival than the Rap-alone group (*P* = 0.0291). In addition to the differences in central memory–dominant T cell phenotypes, the improved metabolic state of CAR-T cells through Met+Rap pretreatment might have contributed to the observed survival advantages.

To ascertain that Met+Rap treatment is effective in both sexes, we conducted a similar survival study using female mice (Supplemental Figure 5A). In this study, all 15 mice receiving CAR-T cells without pretreatment died by day 51 (median survival 38 days). In contrast, 5 out of the 11 mice treated with Met+Rap–pretreated CAR-T cells survived until day 80 (median survival 46 days), demonstrating a significant improvement in survival (*P* = 0.0004). Additionally, using bioluminescence imaging, we observed a reduction in tumor size in the Met+Rap–pretreated group (Figure 4C and Supplemental Figure 5B).

### Mass cytometric analysis reveals the effects of Met+Rap–pretreated CAR-T cells in the glioma microenvironment.

To gain an in-depth understanding of how Met+Rap–pretreated CAR-T cells affect the glioma microenvironment, we evaluated the expression of a panel of metabolic markers (21) in the postinfusion glioma tissue using mass cytometry. C57BL/6J mice bearing intracerebral SB28 mEGFR vIII tumors received an i.v. infusion of 3 × 10^6^ CD45.1^+^ CAR-T cells pretreated with Met, Rap, Met+Rap, or control without pretreatment. Ten days after the CAR-T cell administration, we euthanized the mice and profiled brain-infiltrating leukocytes (BILs) by mass cytometry (Figure 4D). We clustered BILs on the uniform manifold approximation and projection (UMAP) plot, grouped them into 12 subpopulations, and annotated them based on the expression status of lineage markers, glycolysis markers (Glut1, GAPDH, and p-S6), and OXPHOS markers (ATP5a, VDAC1, p-CREB, and cytochrome *c* [CytoC]) (Figure 4, E and F). CD45.1^+^ and CD45.2^+^ BILs were glioma-infiltrating CAR-T cells and hosted C57BL/6J mouse–derived cells, respectively (Figure 4, E and F). We found a significantly higher number of CAR-T cells in the tumors of mice that received Met+Rap–pretreated CAR-T cells than those that received CAR-T cells without pretreatment (Figure 4G). Interestingly, the glioma tissues infiltrated by Met+Rap–pretreated CAR-T cells were infiltrated by significantly fewer Ly6C^+^CD11b^+^ monocytic myeloid-derived suppressor cells (MDSCs) (Figure 4G). These data suggest that Met+Rap–pretreated CAR-T cells have unique abilities to render the glioma microenvironment less immunosuppressive.

### NanoString assay reveals enhanced cytotoxicity and reduced exhaustion of BILs following infusion of Met+Rap–pretreated CAR-T cells.

**Figure 5:**
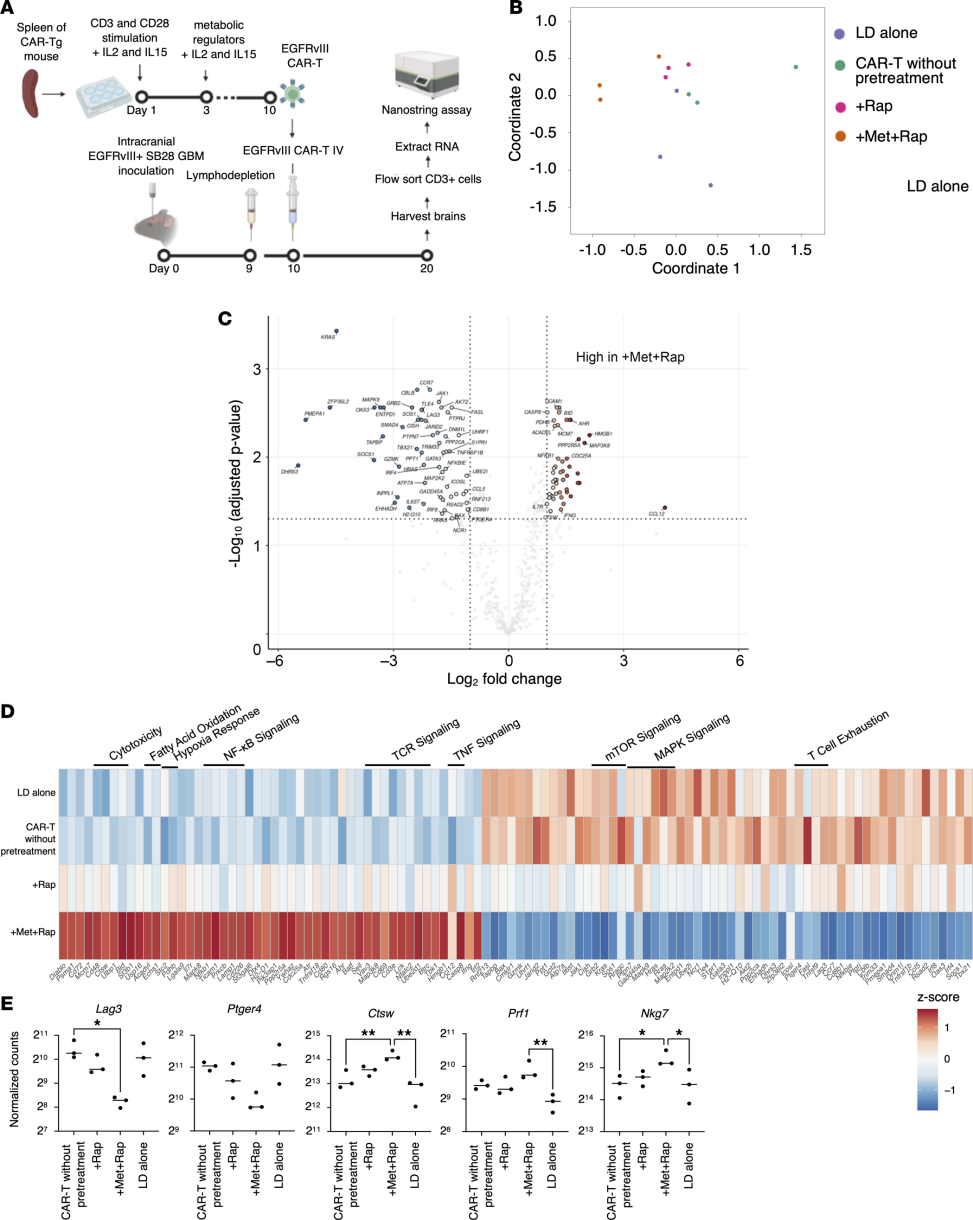
Tumor-infiltrating T cells exhibit enhanced cytotoxic phenotype and lower Lag3 expression in mice that received Met+Rap–pretreated CAR-T cells. (**A**) The design of the experiment. IV, intravenous. (**B**) Multidimensional scaling (MDS) plot colored by the pretreatment types. (**C**) Comparison of 773 genes between CD8^+^ CAR-T cells without pretreatment and Met+Rap–pretreated CD8^+^ CAR-T cells was summarized in volcano plots. (**D**) Genes with |log_2_(fold change)| > 1.0 and –log_10_(adjusted *P* value) > 1.3 were considered significant. Heatmap visualizing the relative expression (*z* score) of genes in each subpopulation. (**E**) Representative gene expression levels obtained from NanoString analysis, highlighting the most distinctive ones. *P < 0.05; **P < 0.01 by 1-way ANOVA followed by Tukey’s multiple-comparison test.

To evaluate the gene expression profile of T cells in the posttreatment tumor microenvironment, we conducted the NanoString assay following an experimental design similar to mass cytometric analyses. Ten days after the i.v. CAR-T cell infusion, we extracted the brains, performed FACS to isolate CD3-positive cells, extracted total RNA, and analyzed the samples using the Mm Exhaustion panel in the NanoString assay with biological triplicates (*n* = 3 mice per group) (Figure 5A). Biological triplicates were used for each group, yielding reliable gene expression reproducibility (Figure 5B). The results comparing the group with no pretreatment and the group with Met+Rap pretreatment are depicted in Figure 5C. Remarkably, T cells from the Met+Rap–pretreated group displayed higher expression levels of genes associated with cytotoxicity, hypoxia response, and T cell receptor (TCR) signaling. In contrast, genes related to mTOR signaling, MAPK signaling, and T cell exhaustion were expressed at higher levels by T cells from the group with no pretreatment. Notably, exhaustion markers, such as *Lag3*, *Pdcd1*, and *Ptger4*, showed a trend toward lower expression levels in the Met+Rap–pretreated group. In contrast, markers of cellular cytotoxicity, such as *Ctsw*, *Prf1*, and *Nkg7*, displayed a trend toward higher expression levels in the Met+Rap–pretreated group (Figure 5, D and E). Although the presence of endogenous T cells limited the detection of significant signal changes, these findings suggest that Met+Rap–pretreated CAR-T cells promote the creation of a more favorable tumor microenvironment.

Furthermore, as shown in Figure 5, C and D, the Met+Rap–pretreated group demonstrated an elevated expression of *Ifng* compared with the untreated CAR-T group. Based on previous studies (22), including ours (23), demonstrating the critical role of T cell–derived IFN-γ in the favorable tumor microenvironment, we treated tumor-bearing mice with CAR-T cells derived from *Ifng*-KO mice (Supplemental Figure 6A). The absence of IFN-γ expression in Met+Rap–pretreated CAR-T cells abolished the reduction in intratumoral MDSCs (Supplemental Figure 6B). These findings suggest a pivotal involvement of the IFN-γ pathway in the diminution of MDSCs in the tumor microenvironment.

### Met+Rap pretreatment is also effective for human CAR-T cells.

**Figure 6:**
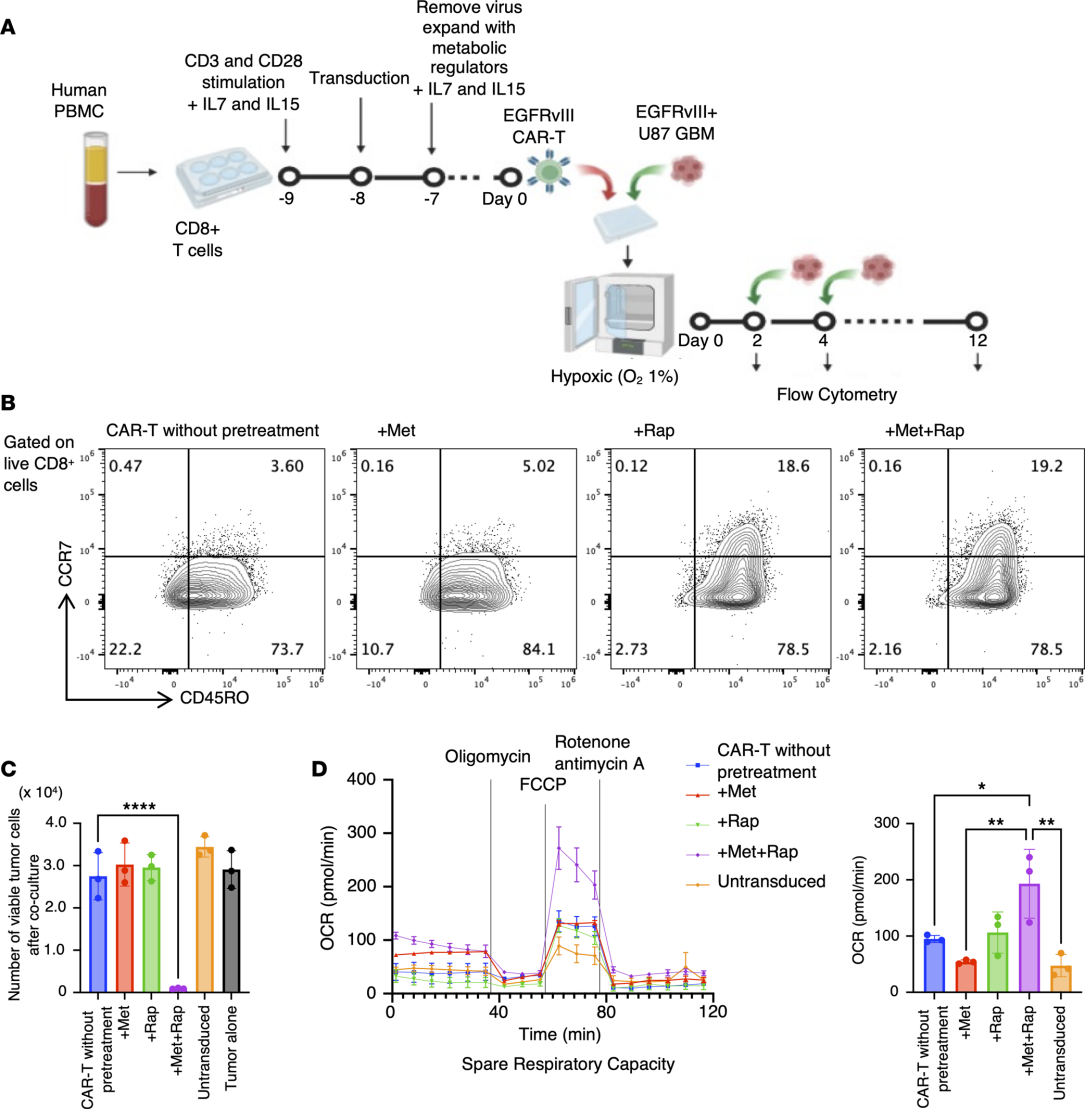
Met+Rap pretreatment enhances the sustained function of human CAR-T cells. (**A**) Experimental design. (**B**) Flow cytometric plots for CD45RO and CCR7 in CD8^+^ CAR-T cells on day 0. (**C**) Graph showing the number of surviving tumor cells after coculture on day 10 under hypoxic condition. Data are presented as mean ± SD. (**D**) Left: OCR of CAR-T cells was measured by Seahorse XFe96 analyzer on day 0. Data are presented as mean ± SEM. Right: Spare respiratory capacity was calculated. Data are presented as mean ± SD. *P < 0.05; **P < 0.01; ****P < 0.0001 by 1-way ANOVA followed by Tukey’s multiple-comparison test.

A series of murine data collectively showed that the Met+Rap pretreatment could maintain the metabolic status and enhance the therapeutic efficacies of murine CAR-T cells. To determine whether metabolic regulators could enhance the therapeutic effectiveness and metabolic state of human CAR-T cells, we treated human anti-EGFRvIII CAR-T cells with Met+Rap during expansion and cocultured them with human GBM U87 EGFRvIII cells under hypoxic conditions (Figure 6A). The human CAR-T cells were generated by sorting CD8-positive cells from PBMCs derived from healthy donors and then transducing them with lentivirus encoding anti-EGFRvIII CAR (Supplemental Figure 7A). Additionally, the administration of metabolic regulators to CAR-T cells did not notably affect the proliferative capacity of human CAR-T cells throughout the expansion phase (Supplemental Figure 7B). Pretreatment with Rapa or Met+Rap resulted in a slight increase in the central memory population in human CAR-T cells, albeit less pronounced than our observations in mice (Figure 6B). Additionally, as illustrated in Figure 6C, the pretreatment of human CAR-T cells with Met+Rap, but none of the other pretreatments, led to persistent and robust antitumor efficacy even following the challenge with tumor cells 5 times under the hypoxic condition. These results were consistent across 3 donors with varying age, sex, and race (Supplemental Figure 7C). Moreover, the Mito Stress Test revealed increased SRC in human CAR-T cells pretreated with Met+Rap, as depicted in Figure 6D, suggesting that this therapy could be effective in human CAR-T cells. Human CD4^+^ CAR-T cells demonstrated similar findings: an increase in central memory T cells, sustained antitumor efficacy under hypoxia, and prolonged cytokine secretion when cocultured with EGFRvIII-positive tumor cells under hypoxic conditions (Supplemental Figure 8, A–D).

## Discussion

In this study, we grappled with the pivotal challenge of enhancing the efficacy of immunotherapy against GBM, an unrelenting brain tumor known for its resistance to conventional treatments. The intricate microenvironment of GBM, characterized by immunosuppression and hypoxic conditions, presents significant obstacles to successful immunotherapy outcomes (5). To surmount these challenges, we delved into the potential of metabolic conditioning to enhance the functional capabilities of CAR-T cells within this complex milieu.

In the context of glioma, tumor-induced vascular endothelial damage is recognized for causing microthromboembolization, leading to hypoxic conditions (24). Additionally, our hypoxyprobe experiments illustrated that immune cells infiltrating intracranial GBM experience a profoundly hypoxic condition compared with those in subcutaneously transplanted GBM. Therefore, the reduced OXPHOS activity observed in the CAR-T cells within the glioma microenvironment is likely due to tissue hypoxia and constrained oxygen availability. These observations led us to hypothesize that preconditioning CAR-T cells with metabolic regulators before infusion would enhance their mitochondrial function, making them more resistant to the induction of exhaustion by the GBM’s hypoxic microenvironment.

Our findings lend strong support to the efficacy and feasibility of this approach. Preconditioning CAR-T cells with the Met+Rap combination significantly enhanced their cytotoxic potency and endurance within the hypoxic environment. While one previous study reported direct antineoplastic effects of Met+Rap combination treatment in vitro, the Met doses used in those studies (10–20 mM) are much higher than those in our research (10 μM) (25). Furthermore, although a previous report indicated that Met negatively impacted the proliferation and cytotoxicity of CAR-T cells (26), this referenced study used much higher doses of Met (1–20 mM). This discrepancy in dosage likely explains the absence of significant inhibitory effects on cell proliferation or diminished antitumor efficacy in our study. An intriguing study in aging research revealed that mice systemically treated with the Met+Rap combination exhibited extended lifespans (27). In a murine Crohn disease model (28), the Met+Rap combination therapy robustly inhibited mTOR signaling and reduced inflammation. Our investigation demonstrated that low-dose ex vivo treatment with Met effectively activated AMPK, while Rap inhibited mTORC1 and mTORC2, leading to efficient activation of PGC-1α in Met+Rap–pretreated CAR-T cells. Consequently, this resulted in an expanded SRC within Met+Rap–pretreated CAR-T cells. These findings were consistent in both murine and human CAR-T cells, highlighting the potential translatability of this approach to human trials.

While prior attempts to enhance T cell metabolism have included both in vitro (29, 30) and in vivo interventions (31), our approach stands out by minimizing the potential of in vivo side effects. This uniqueness rests in our methods solely involving the in vitro expansion phase of CAR-T cell preparation without necessitating in vivo administration of Met or Rap. However, questions linger about the persistence of the improved metabolic states induced in vitro (4). Our study demonstrated that Met+Rap–pretreated CAR-T cells, after chronic stimulation under hypoxic conditions for at least 1 week, maintained their tumor-killing effects and preserved mitochondrial function, supported by Seahorse assay data.

Most significantly, a single i.v. infusion of Met+Rap–pretreated CAR-T cells significantly prolonged the survival of mice bearing intracerebral glioma (median overall survival 49 days in the Met+Rap group vs. 34 days in the non-pretreated CAR-T cell group). Furthermore, our in vivo experiments underscored the clinical relevance of Met+Rap–pretreated CAR-T cells. Besides hypoxia, a notable feature of the brain tumor microenvironment is the abundance of MDSCs (32). In our study, we confirmed by mass cytometry that there was a significant reduction in MDSCs in the brains of mice with gliomas treated with Met+Rap–pretreated CAR-T cells.

Our NanoString analysis confirmed elevated *Ifng* expression levels when CAR-T cells were pretreated with Met+Rap. We evaluated the contribution of IFN-γ secreted by Met+Rap–pretreated CAR-T cells. In those experiments, utilizing Met+Rap–pretreated *Ifng*-KO CAR-T cells failed to reduce MDSCs. Moreover, our NanoString data also revealed a decrease in *Ptger4* expression in the Met+Rap group. *Ptger4* encodes the prostaglandin E2 receptor 4 (EP4), which has been shown to promote the development of MDSCs within tumors, and inhibiting EP4 has been reported to reduce MDSCs (10, 33, 34). These reports, including ours (10), suggest that Met+Rap–pretreated CAR-T cells reprogram the tumor microenvironment by upregulating IFN-γ and inhibiting EP4.

While our study primarily centers on GBM, the implications of our findings may reach far beyond this specific cancer, extending to various solid tumors marked by an immunosuppressive microenvironment. Notably, CAR-T cells have demonstrated limited efficacy in solid tumors compared with their remarkable success in B cell leukemia or lymphoma (35). This diminished efficacy is particularly pronounced in solid tumors characterized by hypoxia (36). By shedding light on this intricate relationship between tumor hypoxic condition and immune response, we open new paths for innovative therapeutic interventions, potentially redefining how we approach GBM and other challenging solid tumors.

In summary, our study advances immunotherapy by introducing metabolic conditioning to amplify CAR-T cell functionality against the intricate challenges posed by GBM. By unraveling our findings’ mechanisms and broader implications, we contribute to ongoing efforts in crafting innovative and effective immunotherapeutic strategies to combat challenging solid tumors.

## Methods

### Sex as a biological variable.

Our study examined male and female animals, and similar findings are reported for both sexes.

### Mice and cells.

C57BL/6J mice were purchased from the Jackson Laboratory (JAX, 000664). Mice were approximately 9–10 weeks old during the experiment and maintained under specific pathogen–free conditions at the Animal Facility at UCSF, per an Institutional Animal Care and Use Committee–approved protocol. The C57BL/6J-background CD45.1^+^ CAR-transgenic mouse strain carrying EGFRvIII-targeting CAR-T cells with a construct as shown in Supplemental Figure 1A was established in our lab (10). We engineered C57BL/6J background transgenic mice to incorporate the anti-EGFRvIII CAR construct, positioned downstream of a Lox-Stop-Lox cassette within the Rosa26 locus. The original CAR knockin (CAR KI) mouse transgene, initially described in Chuntovaet al. (10), incorporates an IRES-GFP sequence downstream of the CAR transgene, flanked by FRT sequences. To mitigate immunogenicity, excision of the GFP sequence from the transgene was pursued. This process involved breeding CAR-KI mice with the B6 Rosa26 FLPo mouse strain (JAX, 012930). Because the CAR transgene was inserted into the Rosa26 locus, half of the offspring exhibited FLPo/CAR KI heterozygosity on each allele at the Rosa26 locus. The excision of GFP was confirmed by PCR genotyping of FLPo/CAR-KI mice. Subsequently, FLPo/CAR-KI mice were bred to remove the FLPo allele until CAR KI/KI homozygosity was achieved. The resultant GFP-excised CAR-KI mice were bred with CD4-Cre transgenic mice (JAX, 022071) and the resulting offspring were genotyped by PCR for expression of both transgenes, enabling the specific expression of the CAR construct in T cells. All mice used for breeding and experiments maintained Cre hemizygosity to minimize any unintended consequences of Cre random insertion. The primer list is available in Supplemental Table 1.

The murine SB28 glioma cell line was established in our lab (37). To create SB28 cells expressing human EGFRvIII, the cells were retrovirally transduced with human EGFRvIII (SB28 hEGFRvIII) as previously described (10). Separately, to generate SB28 cells expressing murine EGFRvIII, we removed the existing GFP reporter gene from SB28 using CRISPR/Cas9 to prepare the cells for subsequent modifications. Those modified SB28 were transduced with a lentiviral vector encoding mouse EGFRvIII and GFP (provided by Yi Fan, University of Pennsylvania, Philadelphia, Pennsylvania, USA) (38). After transduction, GFP-positive cells, which concurrently expressed mEGFRvIII (SB28 mEGFRvIII), were isolated using a cell sorter. Cell lines were cultured in RPMI 1640 medium (Gibco) with 10% (v/v) heat-inactivated FBS and 1% (v/v) penicillin-streptomycin solution (Gibco, 15070063). Cell lines were free of mycoplasma contamination.

### In vitro T cell cultures.

The spleen was harvested to isolate CD3^+^ CAR-T cells from 8- to 12-week-old CAR-T mice. The spleen was minced and treated with ammonium-chloride-potassium (ACK) lysing buffer for 2 minutes to lyse the erythrocytes. CD3^+^ CAR-T cells were then purified from lymphocytes using a MojoSort Mouse CD3 T Cell Isolation Kit according to the manufacturer’s instructions (BioLegend, 480031). CAR-T cells were then activated for 2 days at 1 × 10^6^ cells per well of 24-well flat-bottomed plates with an equivalent number of washed anti-CD3/anti-CD28 Dynabeads (Gibco, 11453D), 30 U/mL human IL-2 (hIL-2) (NIH), and 50 ng/mL mouse IL-15 (mIL-15) (Peprotech, 21015) in 1 mL of complete RPMI (cRPMI: RPMI 1640 media with 10% FBS, 1% penicillin-streptomycin, 1% HEPES [Gibco, 15630080], 1% GlutaMax [Gibco, 35050061], 1% nonessential amino acids [Gibco, 11140076], 1% sodium pyruvate [Gibco, 11360070], and 0.5 mM 2-mercaptoethanol [Gibco, 21985023]). After stimulation, CAR-T cells were cultured for 7–10 days in a medium containing 30 U/mL hIL-2 and 50 ng/mL mIL-15 as an expansion step. Cell density was monitored daily and maintained in fresh media and cytokines at 0.5 × 10^6^ to 1 × 10^6^ T cells/mL. For metabolic regulator pretreatment, 1 × 10^6^ T cells/mL were treated with metabolic regulators at the doses indicated in Table 1 during the expansion step. The medium was changed to a fresh medium containing fresh metabolic regulators every 2 days throughout the pretreatment. The compound names and vendor names of the metabolic regulators are listed in Table 1.

### In vitro coculture.

T cells (0.5 × 10^5^ to 1 × 10^5^ cells) were cocultured with SB28 mEGFRvIII or SB28 hEGFRvIII tumor cells at an effector/target ratio of 1:1 in 96-well flat-bottom plates in triplicate for 2 days in cRPMI containing 30 U/mL hIL-2 and 50 ng/mL mIL-15. After 2 days, cells were collected, washed twice with PBS, and used in FACS analyses. In the FACS data, GFP-positive and Zombie-negative cells were determined to be the surviving tumor cells after coculture (Supplemental Figure 9). Coculture under hypoxia was performed at 1% oxygen using a hypoxic incubator [Binder, CD(E6.1)], as it has been reported that the oxygen concentration in a normal brain is 1%–5% (39, 40). Furthermore, since gliomas are known to be more hypoxic than normal brains, and several reports have observed gliomas with 1% oxygen concentration in vitro, we also used 1% oxygen in this study (41, 42).

### Mouse therapy model.

An aliquot of 5 × 10^3^ SB28 hEGFRvIII or mEGFRvIII cells/mouse was stereotactically injected into the right hemisphere of anesthetized C57BL/6J mice (day 0). Tumor progression was evaluated by luminescence emission on a Xenogen IVIS Spectrum after intraperitoneal injection of 1.5 mg of D-luciferin (GoldBio). Before treatment, mice were randomized, so the initial tumor burden in each group was equivalent. A combination of cyclophosphamide (3–4 mg/mouse) and fludarabine (1 mg/mouse) was intraperitoneally injected as lymphodepletion (LD) around days 10–14. Mice received i.v. administration with 1 × 10^6^ CAR-T cells for survival study the day after LD.

### Isolation of brain tumor-infiltrating immune cells.

For BIL analysis, the brain tumor was harvested and sliced into 3-mm pieces with scalpels, followed by digestion with collagenase type IV (Gibco, 17104019) and deoxyribonuclease I (Worthington Biochemical, LS002007) using a gentleMACS Dissociator (Miltenyi Biotec). Then BILs were isolated using Percoll (Sigma-Aldrich, P1644) as previously described (43).

### Preparation of retroviral vectors.

In our laboratory, we developed the pMSCV-m3C10 vector (10). To produce the virus, we transfected HEK293T cells with this vector using Fugene HD (Promega) as per the manufacturer’s instructions. Forty-eight hours after transfection, we collected the retrovirus-enriched supernatant and filtered it through a 45-μm filter.

### Transduction of mouse T cells by retroviral vectors.

CD3^+^ T cells were extracted from the spleens of 9-week-old C57BL/6J mice and *Ifng*-KO C57BL/6J mice [B6.129S7(B6)-*Ifng^tm1Ts^*/J; JAX, 002287], utilizing the MojoSort Mouse CD3 T Cell Isolation Kit. After isolation, the T cells were activated in 24-well flat-bottom plates, with each well containing 1 × 10^6^ cells. The activation medium consisted of an equal ratio of anti-CD3/anti-CD28 Dynabeads, 30 U/mL hIL-2, and 50 ng/mL mIL-15, all in 1 mL of cRPMI. Concurrently, 6-well non–tissue culture plates were coated with 20 μg/mL RetroNectin (Takara Bio USA, T100B) and refrigerated overnight at 4°C.

On the day designated for transduction, the RetroNectin-coated plates were infused with 0.5 mL of viral supernatant per well and centrifuged at 2,000*g* for 30 minutes at 32°C. Simultaneously, the activated T cells were collected, magnetized to detach the activation beads, rinsed, and then resuspended in a mixture of viral supernatant and polybrene (Sigma-Aldrich; achieving a final concentration of 8 μg/mL) at a density of 2 × 10^6^ cells/mL. Subsequently, 1 × 10^6^ T cells were allocated to each well prefilled with the virus, followed by centrifugation at 1,000*g* for 60 minutes at 32°C. The next morning, the media laden with the virus was delicately removed, and fresh media supplemented with cytokines (30 U/mL hL-2, 50 ng/mL mIL-15) were introduced. For the pretreatment with metabolic regulators, T cells at 1 × 10^6^ cells/mL were treated with the designated metabolic regulators as per the concentrations outlined in Table 1, starting the day after transduction. The culture medium, inclusive of fresh metabolic regulators, was replenished every 2 days throughout the pretreatment period.

### Preparation of lentiviral vectors.

The pELNS-3C10-CAR vector was established in our lab (44). HEK293TN cells (5 × 10^5^) were plated on a 6-well plate. At 24 hours, pELNS, pAX, and pMD2.G were cotransfected using Fugene HD Transfection Reagent (Promega). The supernatant was collected at 48 hours and stored at –80°C.

### Human T cell isolation.

Human healthy donor-derived whole blood was obtained from StemExpress (catalog LE005F). Then, PBMCs were isolated by Ficoll density gradient centrifugation. Human CD8^+^ T cells and CD4^+^ T cells were isolated by negative selection using the EasySep Human CD8 negative isolation kit and EasySep Human CD4 negative isolation kit, respectively (StemCell Technologies).

### Transduction of human T cells by lentiviral vectors.

The isolated T cells (1 × 10^6^) were resuspended in 1 mL medium per well of a 24-well plate and stimulated with Dynabeads Human T-Activator CD3/CD28 (Thermo Fisher Scientific), IL-7 (Peprotech, 5 ng/mL), and IL-15 (Peprotech, 5 ng/mL) for 24 hours. Next, the cells were resuspended in 0.5 mL medium mixed with 0.5 mL lentiviral pELNS-3C10-CAR vector supernatant with polybrene (8 μg/mL) and plated on a 24-well plate precoated with RetroNectin and spun at 1,000*g* for 1 hour at 32°C. T cells were then cultured with the lentiviral vector, and the virus and Dynabeads were removed after 24 hours. Cell lines were cultured in X-VIVO medium (Lonza) with 5% (v/v) gamma-irradiated human AB serum (GeminiBio), 10 mM *N*-acetyl-L-cysteine (Sigma-Aldrich), and 50 μM 2-mercaptoethanol (Gibco, 21985023).

### Cytokine assays.

IFN-γ and TNF levels in the culture supernatant from coculture experiments were quantified using enzyme-linked immunosorbent assay (ELISA) kits. Specifically, the Human IFNγ DuoSet ELISA (R&D Systems, DY285B) and Human TNF-alpha Quantikine ELISA Kit (R&D Systems, DTA00D) were utilized for this purpose. All procedures were performed according to the manufacturer’s instructions.

### Flow cytometric analysis.

The following monoclonal antibodies (mAbs) were used to detect the respective antigens in the mouse sample: CD45 (clone 30-F11), CD45.2 (clone 104), CD45.1 (clone A20), CD8 (clone 53-6.7), CD4 (clone RM4-5), CD3 (clone 17A2), CD11b (clone M1/70), Ly6C (clone HK1.4), CD44 (clone IM7), and CD62L (clone MEL-14) from BioLegend; and Glut1 (clone EPR3915) from Abcam. ATP5a expression was detected by anti-ATP5a (Abcam, ab110273), followed by secondary staining with goat anti–mouse IgG2b heavy chain (Abcam, ab130790). The following mAbs were used to detect the respective antigens in the human sample: CD45 (clone HI30), CD8 (clone HIT3a), CD4 (clone RPA T4), CD3 (clone HIT3a), and CD45RO (clone UCHL1) from BioLegend; and CCR7 (clone 3D12) from Invitrogen. In the case of staining for CAR, phycoerythrin-labeled (PE-labeled) Human EGFRvIII Protein, His Tag (Acro Biosystems, EGI-HP2E3) was used. Live/dead cell discrimination was performed using a Zombie Aqua Fixable Viability Kit (BioLegend, 423102). Intracellular staining was performed using a FOXP3 Fixation Kit (eBioscience). All flow cytometry experiments were performed on the Invitrogen Attune NxT (Thermo Fisher Scientific) flow cytometer and analyzed using FlowJo software (FLOWJO, LLC). Data were gated on live (Zombie negative) and single cells.

### In vivo hypoxic condition analysis.

C57BL/6J mice received a stereotactic injection of 1 × 10^4^ SB28 mEGFRvIII cells in the right cerebral hemisphere or subcutaneous injection of 1 × 10^5^ SB28 mEGFRvIII cells mixed 1:1 with Matrigel (Corning, 354230) in the right flank (day –16). LD was performed on day –1, and 3 × 10^6^ CAR-T cells were infused i.v. on day 0. Mice received i.v. pimonidazole (80 mg/kg, hypoxyprobe, hp11) in PBS 1.5 hours before euthanizing on day 5. Pimonidazole was visualized with anti-pimonidazole antibodies (hypoxyprobe, hp11) using a FOXP3 Fixation Kit.

### Seahorse metabolic assays.

The oxygen consumption rate (OCR) of treated cells was measured using a Seahorse XFe96 Extracellular Flux Analyzer (Agilent). One day before the experiment, the XFe96 plate was coated with CellTak solution (Corning, 354240) per the manufacturer’s recommendation. On the day of the experiment, 2 × 10^5^ to 3 × 10^5^ CD8^+^ T cells per well were seeded in the CellTak-coated XFe96 plate, and the OCR was measured. In the Mito Stress Test, oligomycin (2 μM), carbonyl cyanide *p*-trifluoromethoxyphenylhydrazone (FCCP) (2 μM), and rotenone/antimycin A (0.5 μM) were injected to obtain maximal and control OCR values. Different parameters from the OCR graph were calculated. Basal oxygen consumption was defined as follows: (the last rate measurement before oligomycin) – (non-mitochondrial respiration). Maximal respiration was defined as follows: (maximum rate measurement after FCCP) – (non-mitochondrial respiration). SRC was calculated by subtracting basal respiration from maximal respiration.

### Western blotting.

CD8^+^ T cells were isolated from in vitro–treated CAR-T cells using a Mojosort Mouse CD8 T Cell Isolation Kit (Biolegend, 480035). After washing cells with PBS twice, CAR-T cells were lysed in M-PER buffer (Thermo Fisher Scientific, 78501) with protease inhibitors (MilliporeSigma, 11836170001) and phosphatase inhibitors (MilliporeSigma, 4906845001). After measurement of protein concentration by BCA assay (Thermo Fisher Scientific, 23227), a total of 10–20 μg protein was prepared and loaded onto 4%–20% gradient Mini-PROTEAN TGX gels (Bio-Rad, 4561093) and electrophoretically separated at 135 V. Proteins in the gels were transferred to PVDF membranes using the Trans-Blot Turbo Kit and the Trans-Blot Turbo transfer system (Bio-Rad, 1704156). Membranes were then incubated in a blocking buffer (Nacalai Tesque, 13779-01) for 20 minutes at room temperature, followed by incubation with primary antibody overnight at 4°C in the blocking buffer. After washing, secondary antibody incubations were done at room temperature for 60 minutes in the blocking buffer. Blots were developed with enhanced ECL Western Blotting Substrate (Thermo Fisher Scientific, 32106). Primary antibodies recognizing the following proteins were obtained from Cell Signaling Technology: AMPKα (catalog 2532), p-AMPKα (catalog 2535), p70 S6 kinase (P-S6K) (catalog 9202), p-P-S6K (Thr389, catalog 9205), pan-Akt (catalog 4691), p-Akt (Ser473, catalog 9271), S6 ribosomal protein (catalog 2217), p-S6 ribosomal protein (Ser240/244), 4E-BP1 (catalog 9644), and p-4E-BP1 (Thr37/46, catalog 2855). An antibody recognizing PGC-1α (catalog 66369-1-Ig) was obtained from Proteintech. During the detection of PGC-1α, Can Get Signal immunoreaction enhancer solutions (TOYOBO, NKB-101 and NYPBR01) were used instead of a blocking buffer. The densities of immunoreactive bands were quantified using ImageStudioLight software (LI-COR Biosciences).

### NanoString gene expression analysis.

Ten days after CAR-T administration, mice bearing intracerebral SB28 mEGFRvIII tumors were euthanized, and brains were harvested and processed. Mice were treated as groups of 6 mice each, and BILs from 2 mice were pooled as 1 sample so that 3 biological replicates were obtained for each group. We further isolated the CD3^+^ cells by FACSAria (BD Biosciences) and used the purified immune cells for RNA extraction. Total RNA was extracted from flow-sorted CD3^+^ cells using the RNeasyPlus Micro Kit (Qiagen, 74034), following the manufacturer’s instructions. RNA purity and integrity were determined with an RNA 6000 Pico Kit (Agilent, 5067-1513) on a Bioanalyzer 2100 (Agilent).

The cDNA products were amplified for 8 cycles using the nCounter Low RNA Input Kit (NanoString) based on the manufacturer’s protocol. Briefly, 4 μL of RNA (0.78 ng/μL) was used for the cDNA conversion, and the Mm Exhaustion Low-input Primers were used for the cDNA amplification. The cDNA was then incubated at 95°C for 2 minutes. A total of 7.5 μL of the amplified cDNA product was used for the analysis and the nCounter Mm Exhaustion Panel was used. The hybridization reaction was performed for 18 hours at 65°C. After hybridization, samples were loaded on an nCounter SPRINT cartridge and processed on the nCounter SPRINT Profiler. All data normalization was performed using nSolver 4.0 software (NanoString) and ROSALIND (ROSALIND, Inc.), as recommended by the manufacturer.

### Data acquisition for mass cytometry.

Ten days after CAR-T administration, mice bearing intracerebral SB28 mEGFRvIII tumors were euthanized, and brains were harvested and processed. Mice were treated as groups of 12 mice, and BILs from 4 mice were pooled together as 1 sample so that 3 samples were obtained for each group. However, mice that received Rap-treated CAR-T cells had fewer BILs and thus had only 2 samples. Cryopreserved BILs were thawed 1:10 in thawing media (cRPMI + 25 U/mL benzonase). Cells were incubated in 5 mM cisplatin (Cell-ID Cisplatin; Fluidigm), allowing for the distinguishing of live cells. Cells were then fixed with 1.6% paraformaldehyde and barcoded with the Cell-ID 20-Plex Pd Barcoding Kit (Fluidigm). After Fc blocking (TruStain FcX; BioLegend), cells were stained with a metal-conjugated surface antibody cocktail (Table 2). Cells were then permeabilized with a FOXP3 Fixation Kit and stained with an intracellular antibody cocktail (Table 2), followed by resuspension in iridium intercalator (Cell-ID Intercalator; Fluidigm) solution overnight. Cells were then washed and resuspended in a running buffer consisting of a 1:10 dilution of normalization beads (EQ Four Element Calibration Beads; Fluidigm) in deionized water. Samples were then acquired on the Fluidigm Helios Mass Cytometer, and resultant data were exported to FCS files for further processing.

### Processing of mass cytometry data.

Raw FCS files were processed by the normalizer function provided by the Parker Institute of Cancer Immunotherapy Premessa package in R Studio (https://posit.co/downloads/). Normalization bead removal and de-barcoding were performed on the same platform. Each immune subpopulation, such as CD3^+^ T cells, was gated and exported using FlowJo software. These exported files were then uploaded to the Cytofkit2 package, where immune cells were processed using the dimension-reducing algorithm t-SNE or UMAP for visualization in 2D space and clustered using FlowSOM. Standard settings were utilized (with *k* = 15). The cells in each cluster were then phenotyped and analyzed using *z* score–normalized marker expression and population data, respectively. All analytic outputs were generated in R Studio and Prism (GraphPad Software).

### Statistics.

A comparison of survival curves between 2 groups was evaluated by the log-rank test. Variations in data were evaluated as the mean ± standard deviation (SD) or ± standard error of the mean (SEM). R software (version 4.2.1) and Prism software (version 9) were used for data management and statistical analyses. Significance levels were set at 0.05 for all tests.

### Study approval.

All animal studies strictly followed UCSF institutional guidelines (Laboratory Animal Resource Center approval no. AN185402-02).

### Data and materials availability.

All data associated with this study are available in the main text or in the supplemental Supporting Data Values file.

## Author contributions

RH and HO designed the research. RH, KK, AY, TC, SP, and PC conducted experiments. RH, KK, TN, and LSL were involved in the analysis and interpretation of data. MHS and HO provided overall study supervision. RH, KK, AY, TN, LSL, MHS, and HO wrote the manuscript.

## Supplementary Material

Supplemental data

Unedited blot and gel images

Supporting data values

## Figures and Tables

**Table 2:**
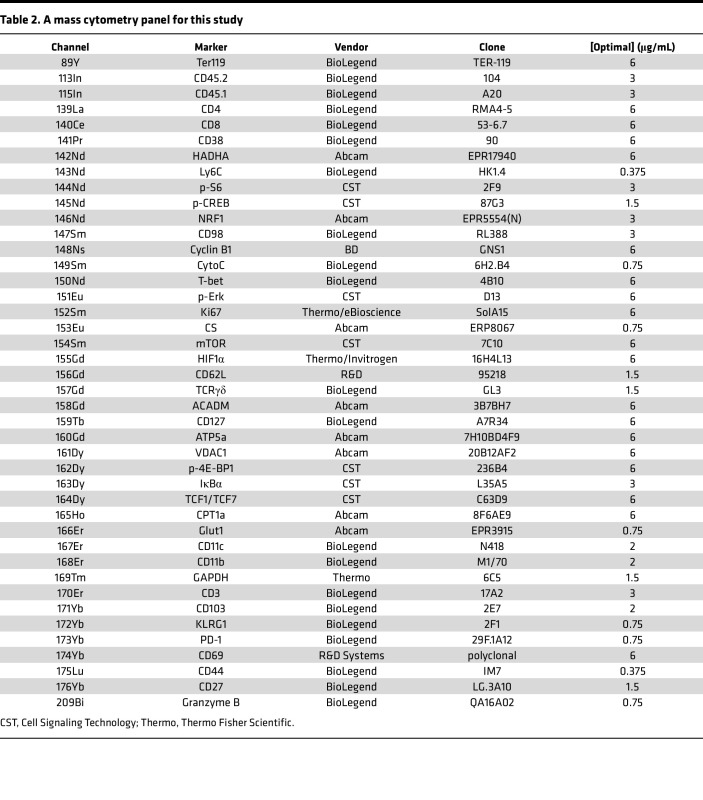
A mass cytometry panel for this study

**Table 1:**
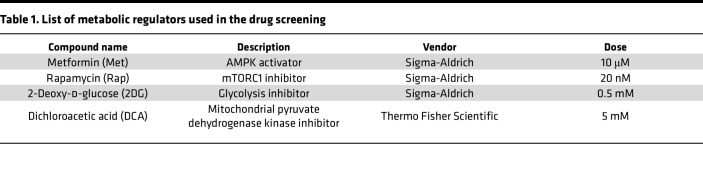
List of metabolic regulators used in the drug screening

## Version 1 02/22/2024

In-Press Preview

## Version 2 04/08/2024

Electronic publication
