# Supplementary material for: Enhancing CAR-T cell metabolism to overcome hypoxic conditions in the brain tumor microenvironment
Source: JCI Insight. 2024 Feb 22;9(7):e177141. doi: 10.1172/jci.insight.177141 (PMC11128202; doi:10.1172/jci.insight.177141)
Supplement: Supplemental data [file jciinsight-9-177141-s009.pdf]

Supplementary Figure 1

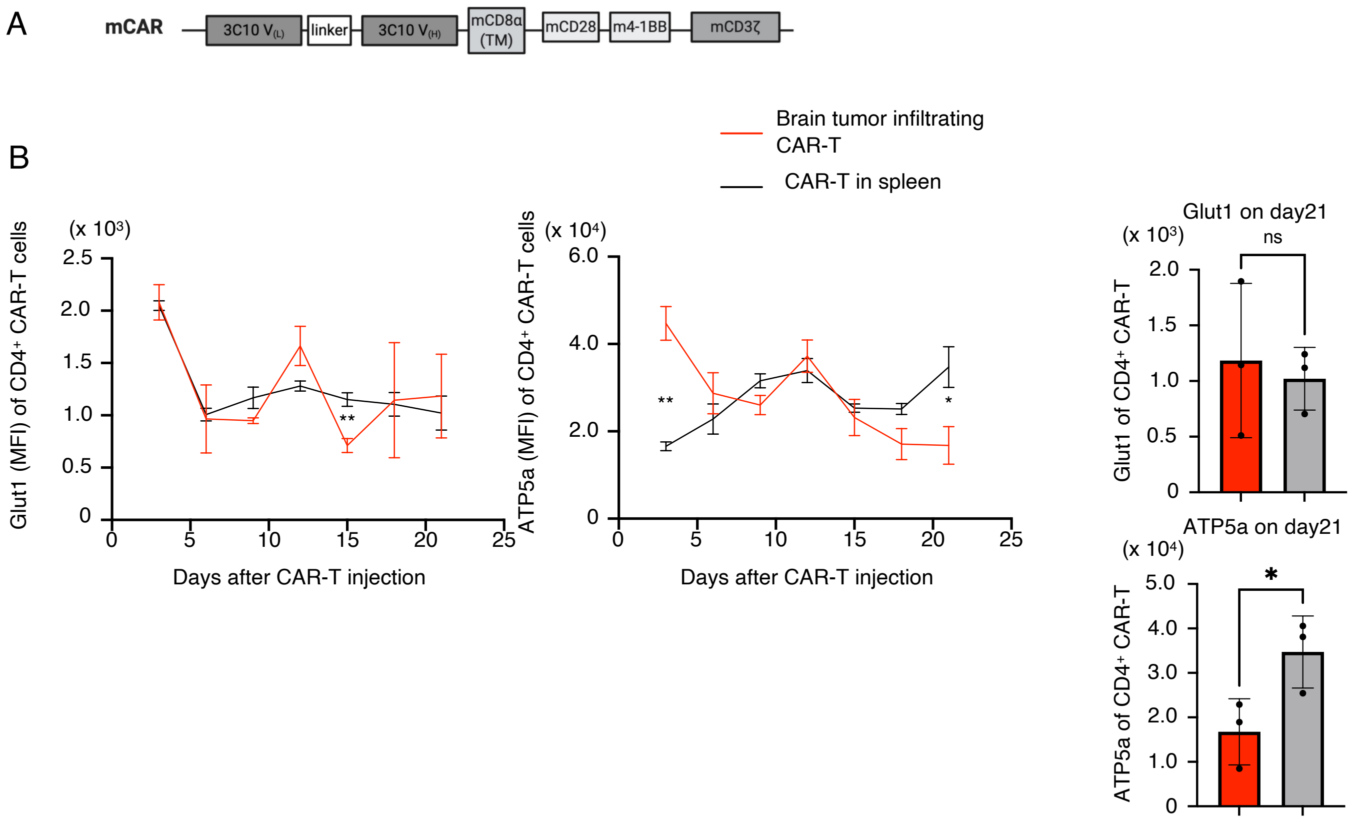

Supplementary Figure 1. Exhaustion of CD4+ CAR-T cells associated with reduced OXPHOS activity in the hypoxic glioma microenvironment.

(A) The structure of CAR-T cells.

(B) Longitudinal changes in Glut1 and ATP5a in glioma-infiltrating CD4+ CAR-T cells (left panels). Expression of Glut1(right top) and ATP5a (right bottom) by mean fluorescence intensity (MFI) in CD4+ CAR-T cells extracted from the spleen (grey) and tumor (red) on Day 21. Error bars show the mean with SD. *$P < 0.05$, **$p < 0.01$ by Unpaired T test.

Supplementary Figure 2

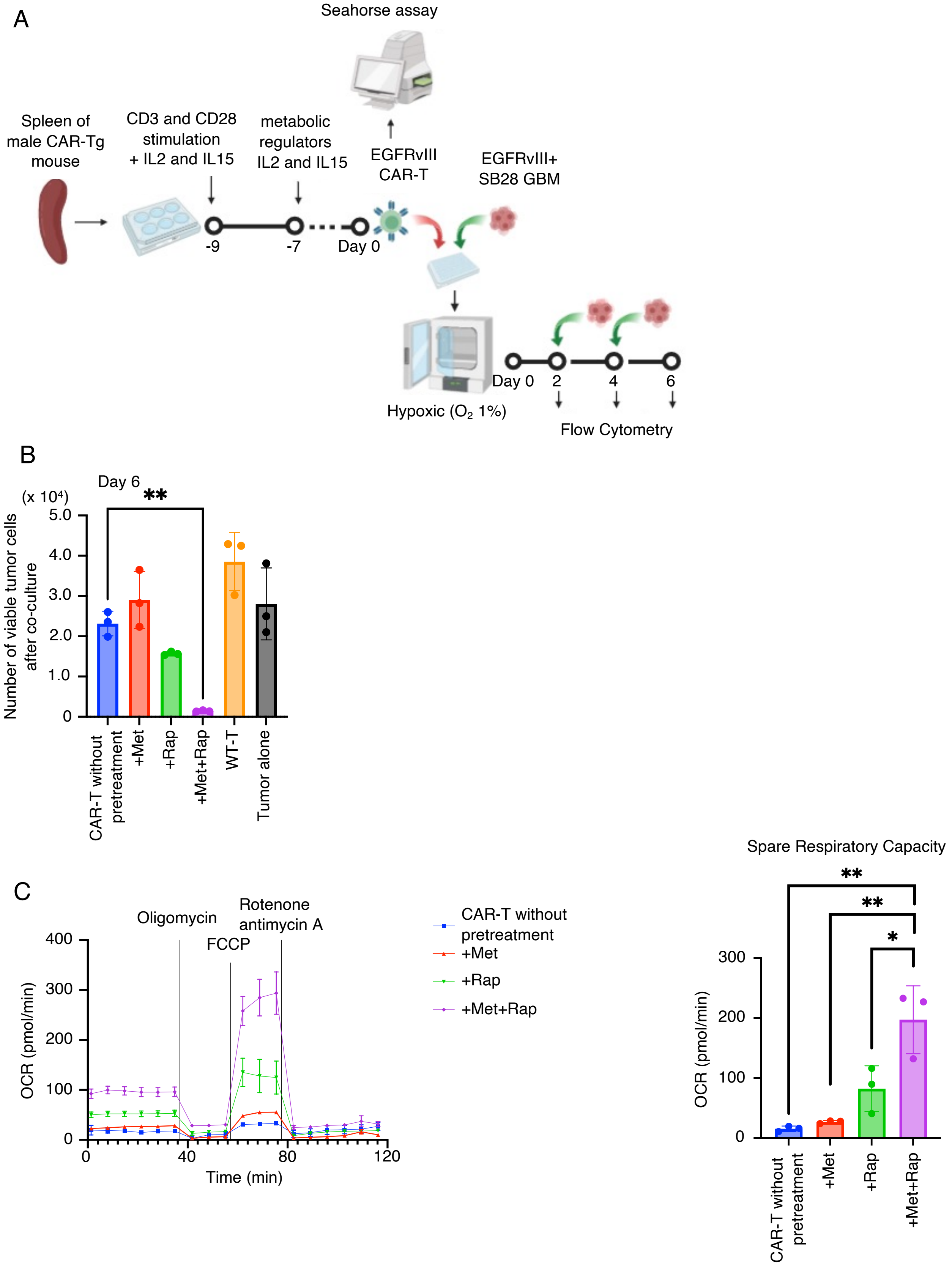

Supplementary Figure 2. Met+Rap is effective on CAR-T cells derived from male CAR-Tg mice.
(A) Experimental design using CAR-T cells derived from male CAR-treated mice.
(B) The number of viable tumor cells on Day 6. Error bars show the mean with SD. $^{**}p < 0.01$ by one-way ANOVA test followed by Tukey's multiple comparisons test.
(C) OCR of CAR-T cells was measured by Seahorse Xfe96 analyzer on Day 0. Data represent the means ± SEM. Spare Respiratory Capacity was calculated (right). Error bars show the mean with SD. $^{*}p < 0.05$, $^{**}p < 0.01$, $^{***}p < 0.001$ by one-way ANOVA test followed by Unpaired T test.

Supplementary Figure 3

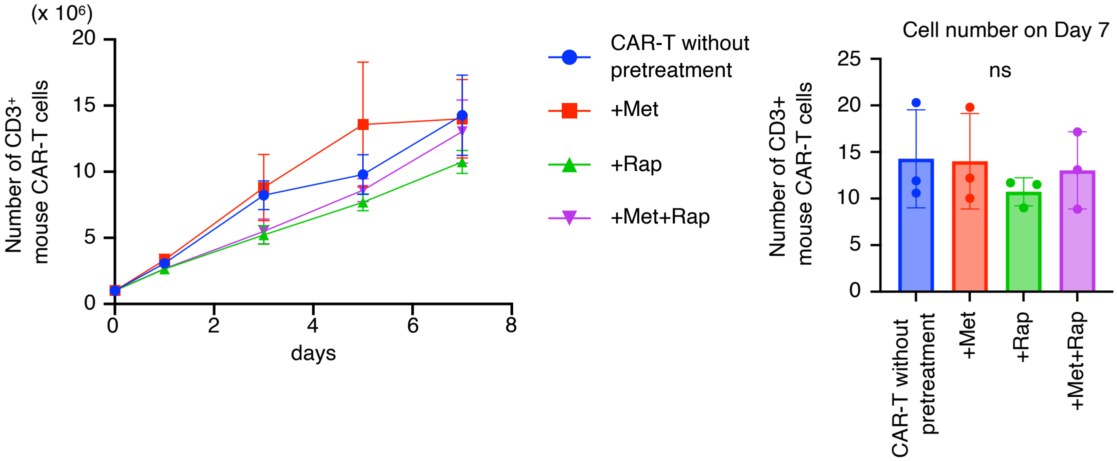

Supplementary Figure 3. Pretreatment of metabolic regulators does not significantly affect the expansion of mouse CAR-T cells.

The line graph illustrates the changes in cell numbers of mouse CD3+ CAR-T cells during in vitro expansion for each pretreatment group (left). The bar graph shows the comparison of cell numbers among different groups after 7 days of culture (right). The line graph displays data as means ± SEM. Error bars on the bar graph show the mean with SD. ns (not significant) by one-way ANOVA test followed by Tukey's multiple comparisons test.

Supplementary Figure 4

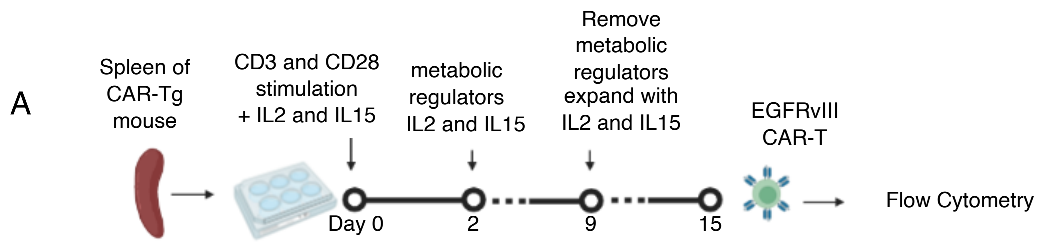

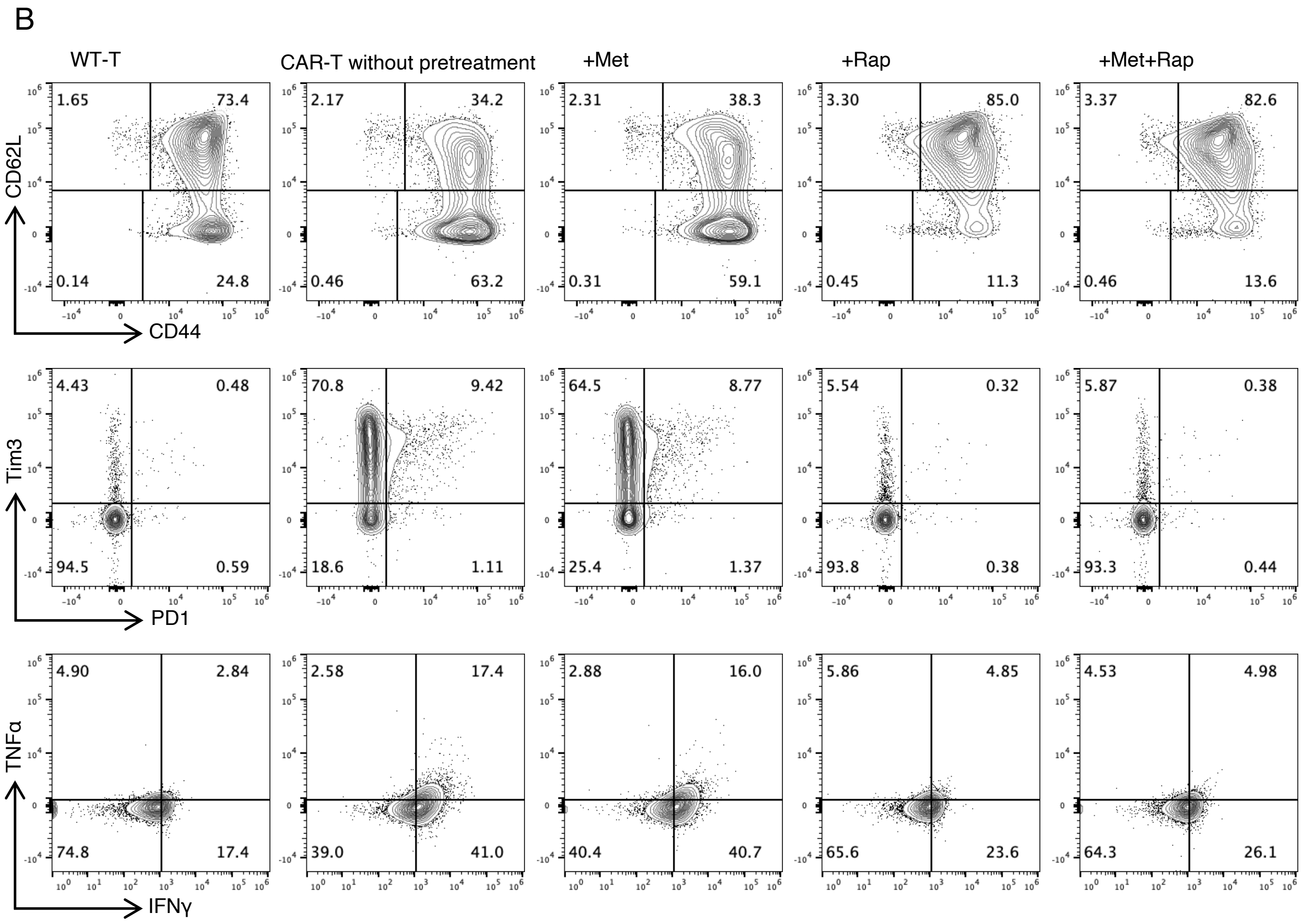

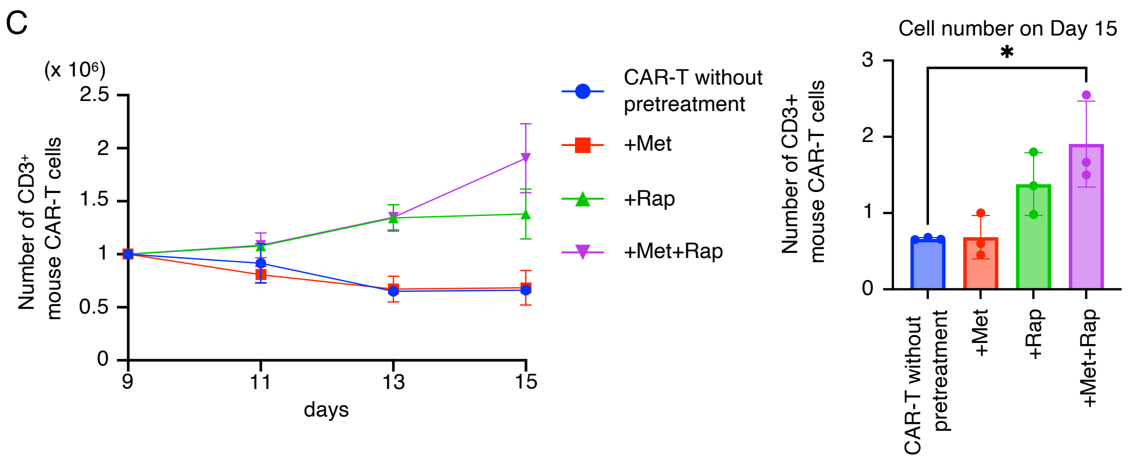

Supplementary Figure 4. Pretreatment with metabolic regulators preserves the central memory phenotype and prevents the exhaustion of CAR-T cells.

(A) Experimental design.

(B) Flow cytometric plots for CD44 and CD62L (upper panels), PD1 and Tim3 (middle panels), and IFNγ and TNFα (lower panels) in CD8+ cells on Day 15.

C) The line graph represents the changes in cell counts for each group during culture under hypoxic condition(left). The bar graph shows the comparison of cell numbers among different groups after 6 days of culture under hypoxic condition(right). The line graph displays data as means ± SEM. Error bars on the bar graph show the mean with SD. *$p < 0.05$ by one-way ANOVA test followed by Tukey's multiple comparisons test.

Supplementary Figure 5

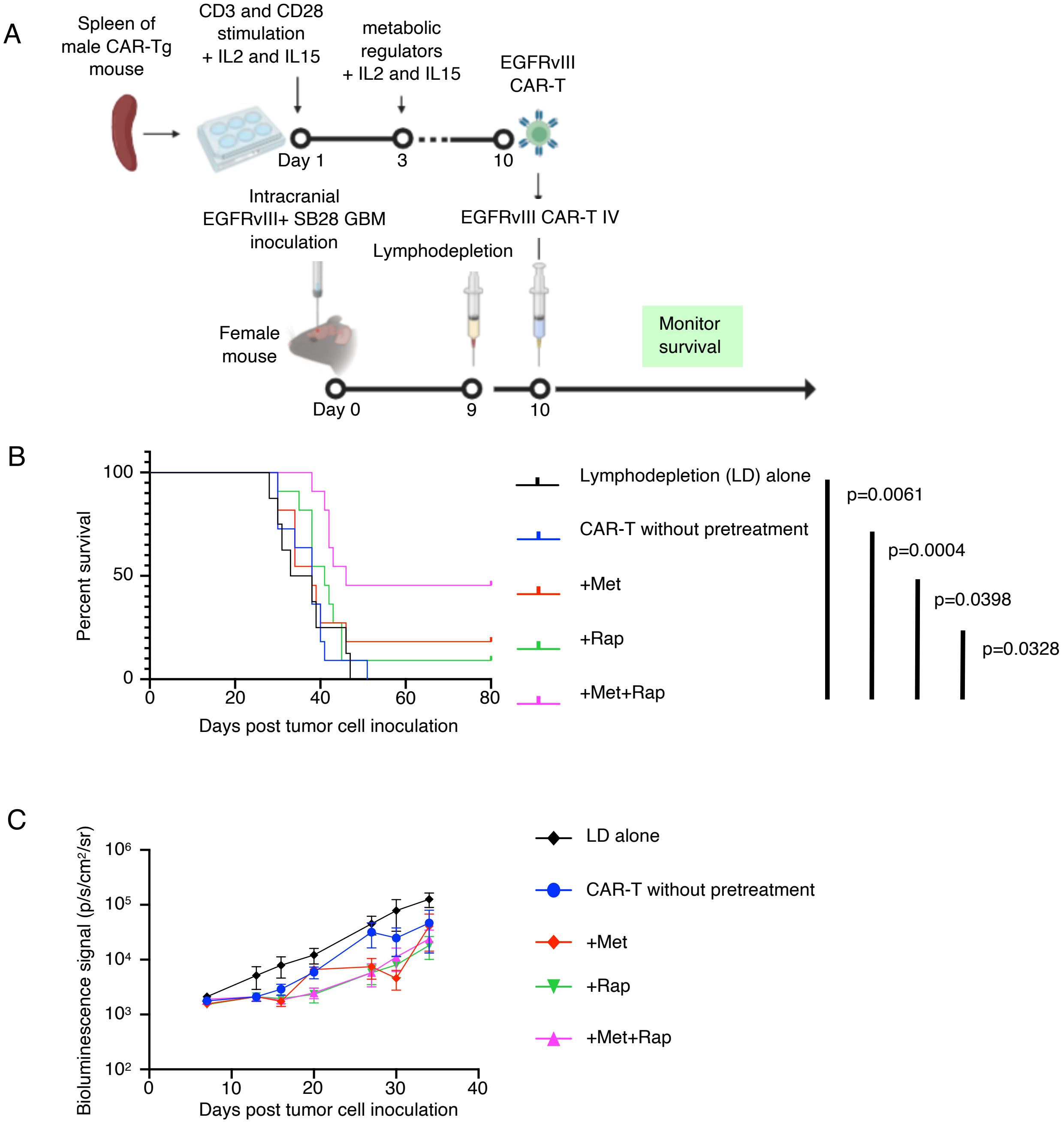

Supplementary Figure 5. Pretreatment of CAR-T cells with Met+Rap extended the survival of glioma-bearing female mice.

(A) Schematic of the treatment protocol for the survival study using male mice.

(B) Kaplan-Meier curves: LD group (MS = 35.5 days, n = 8), CAR-T cells without pretreatment (MS = 38 days, n = 11), +Met-pretreated CAR-T (MS = 38 days, n = 11), +Rap-pretreated CAR-T (MS = 41 days, n = 11), and +Met+Rap-pretreated CAR-T (MS = 46 days, n = 11).

(C) Tumor size was measured by luciferase bioluminescence imaging over time as the number of photons per second per square centimeter per steradian (p/s/cm2/sr). Data represent the means ± SEM.

Supplementary Figure 6

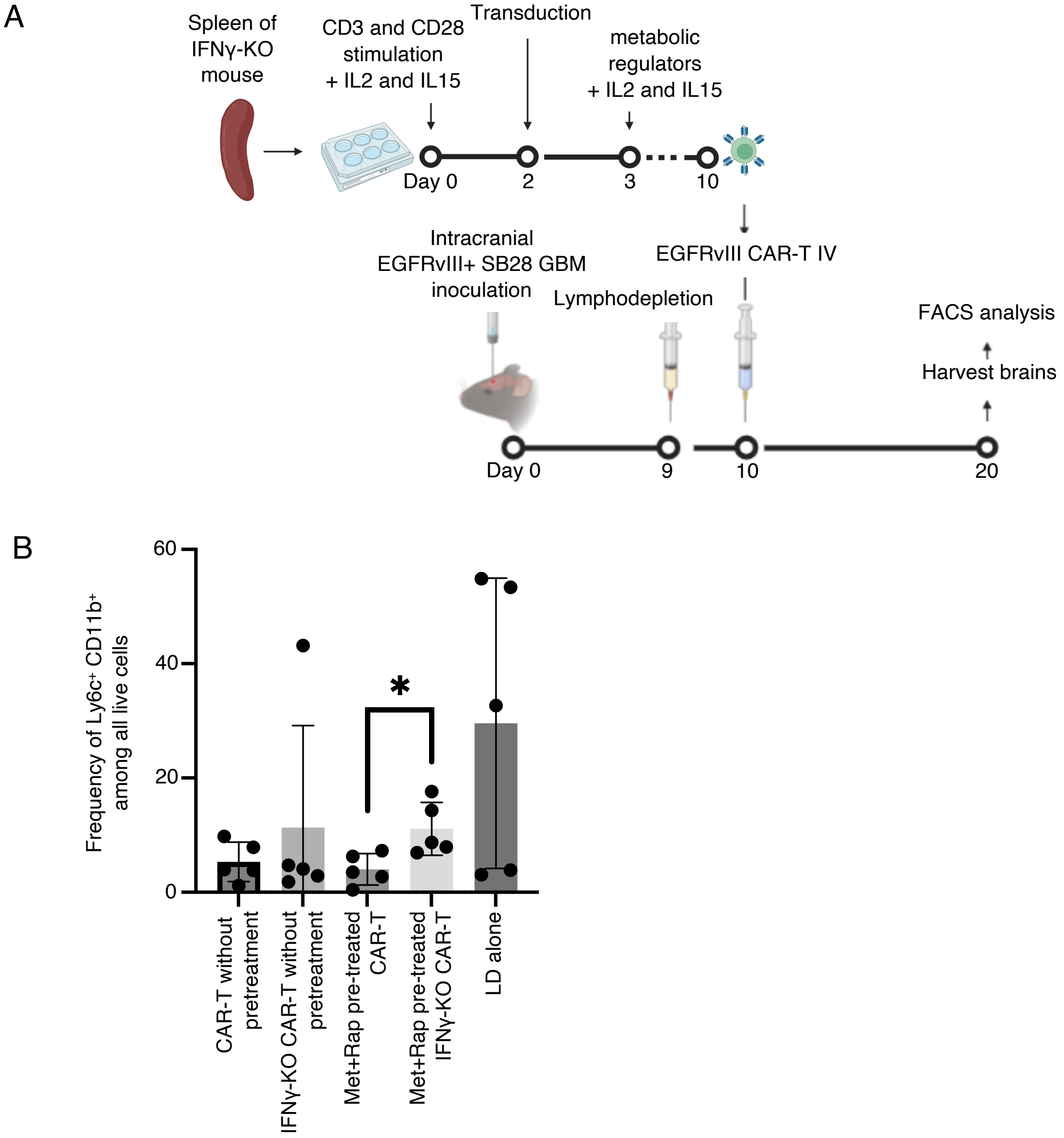

Supplementary Figure 6. Lack of MDSC reduction in glioma by Met+Rap-pretreated CAR-T cells from IFNγ-KO mice.

(A) Experimental design.

(B) Frequencies of SB28 mEGFRvIII-infiltrating Ly6c+ CD11b+ MDSCs among the pretreatment types. Error bars show the mean with SD. *$P < 0.05$ by Unpaired T test.

Supplementary Figure 7

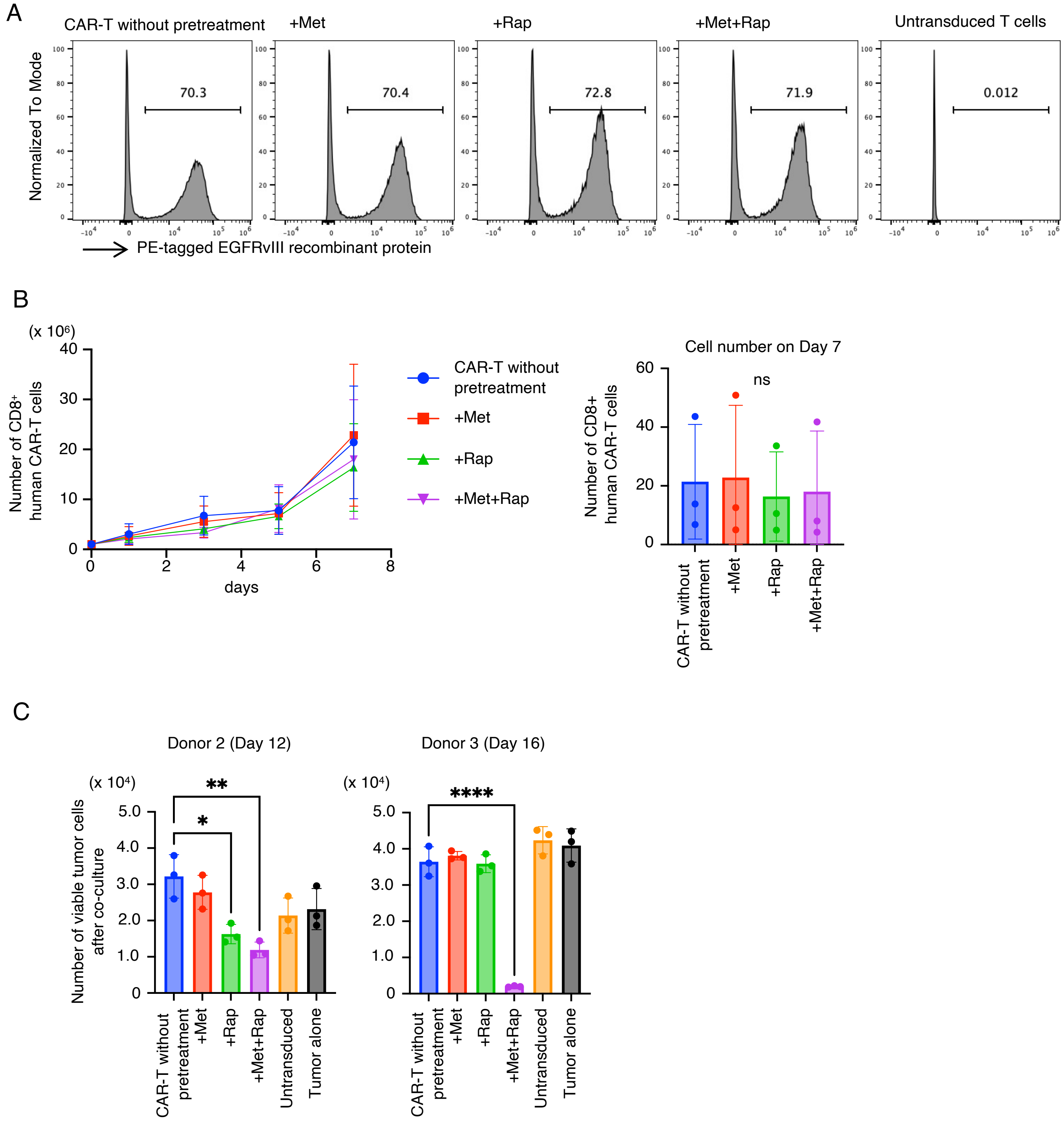

Supplementary Figure 7. The effects of Met+Rap were confirmed in CAR-T cells derived from three donors.

(A) Flow cytometric plots for the expression of anti-EGFRvIII CAR detected by PE-tagged recombinant EGFRvIII protein in CD8+ cells before starting the coculture.

(B) The line graph illustrates the changes in cell numbers of human CD8+ CAR-T cells during in vitro expansion for each pretreatment group (left). The bar graph shows the comparison of cell numbers among different groups after 7 days of culture (right). The line graph displays data as means ± SEM. Error bars on the bar graph show the mean with SD. ns (not significant) by one-way ANOVA test followed by Tukey's multiple comparisons test.

(C) Graph showing the number of surviving tumor cells after long-term coculture under hypoxic condition with CAR-T cells derived from the second and third donors. Error bars show the mean with SD. *$p < 0.05$, ****$p < 0.0001$ by one-way ANOVA test followed by Tukey's multiple comparisons test.

Supplementary Figure 8

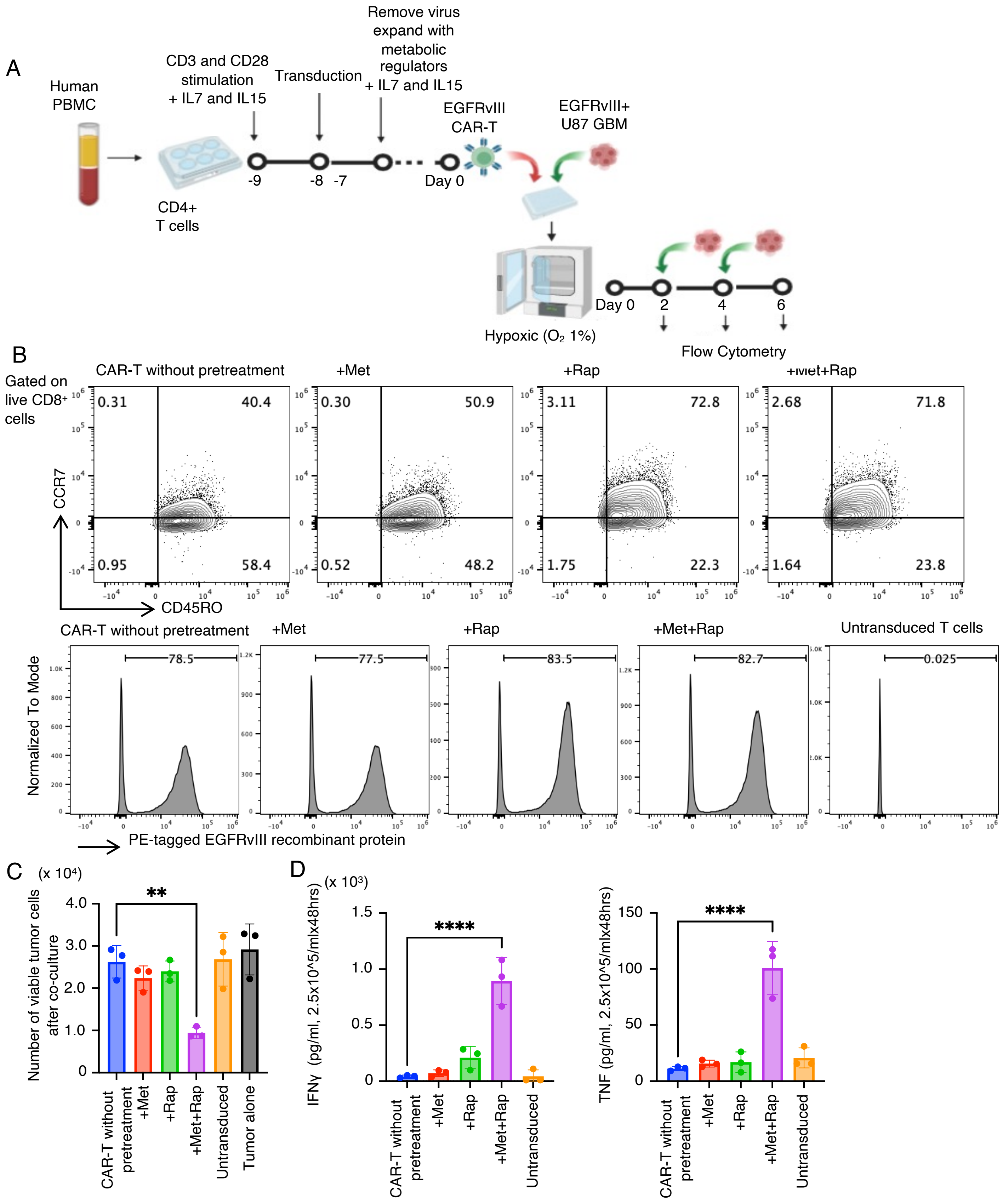

Supplementary Figure 8. Met+Rap pretreatment enhances the sustained function of human CD4+ CAR-T cells.
(A) Experimental design.
(B) Flow cytometric plots for CD45RO and CCR7 and the expression of anti-EGFRvIII CAR in CD4+ CAR-T cells on Day 0.
(C) Graphs showing the number of surviving tumor cells after coculture on Day 6 under hypoxic condition. Error bars show the mean with SD. $^{**}p < 0.01$ by one-way ANOVA test followed by Tukey's multiple comparisons test.
(D) Production of IFNγ and TNF by CD4+ CAR-T cells when cocultured with EGFRvIII expressing U87 tumor cells. Error bars show the mean with SD. $^{****}p < 0.0001$ by one-way ANOVA analysis followed by Tukey's multiple comparison test.

Supplementary Figure 9

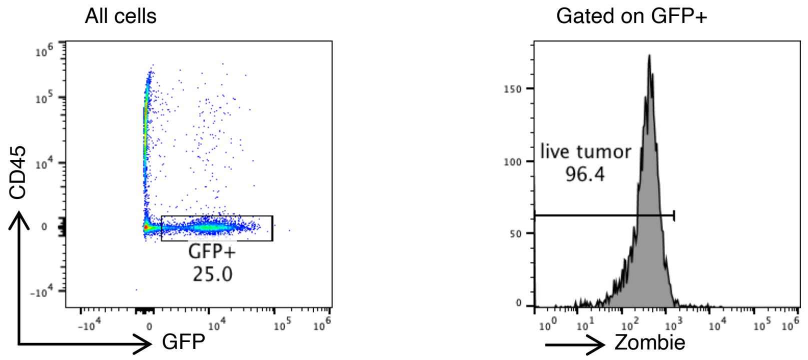

Supplementary Figure 9. A flow cytometry gating strategy was used to define surviving tumor cells after coculture.

Tumor classification was assigned to GFP-positive cells within the CD45-negative subgroup. Cells positive for the Zombie were identified as dead cells and consequently eliminated.

Supplementary Table 1

Primer name	Sequence 5' – 3'	Used for; direction
Rosa_5bisF	GCTCTCCCAAAGTCGCTCTG	mCAR PCR, common; forward
PreCAG2-Rev	CCACTGGAAAGACCGCGAAGAG	mCAR PCR, Tg; reverse
Rosa3ArmR	ACCAGGTTAGCCTTTAAGCC	mCAR PCR, ROSA26; reverse
CD4creF	GTTCTTTGTATATATTGAATGTTAGCC	CD4-cre, common; forward
CD4creMUT	CTTTGCAGAGGGCTAACAGC	CD4-cre, Tg; reverse
CD4creWT	TATGCTCTAAGGACAAGAATTGACA	CD4-cre, WT allele; reverse
FrtGFP-FWD	TGCAAACCTCCAGGACCCTA	EGFP allele in mCAR Tg; forward
FrtGFP-REV	TGGCTGGCAACTAGAAGGC	EGFP allele in mCAR Tg; reverse

Supplementary Table 1.
Primer sequences utilized for generating CAR transgenic mice.
