# Supplementary material for: Enhancing CAR-T cell metabolism to overcome hypoxic conditions in the brain tumor microenvironment
Source: JCI Insight. 2024 Feb 22;9(7):e177141. doi: 10.1172/jci.insight.177141 (PMC11128202; doi:10.1172/jci.insight.177141)
Supplement: Unedited blot and gel images [file jciinsight-9-177141-s010.pptx]

## Slide 1
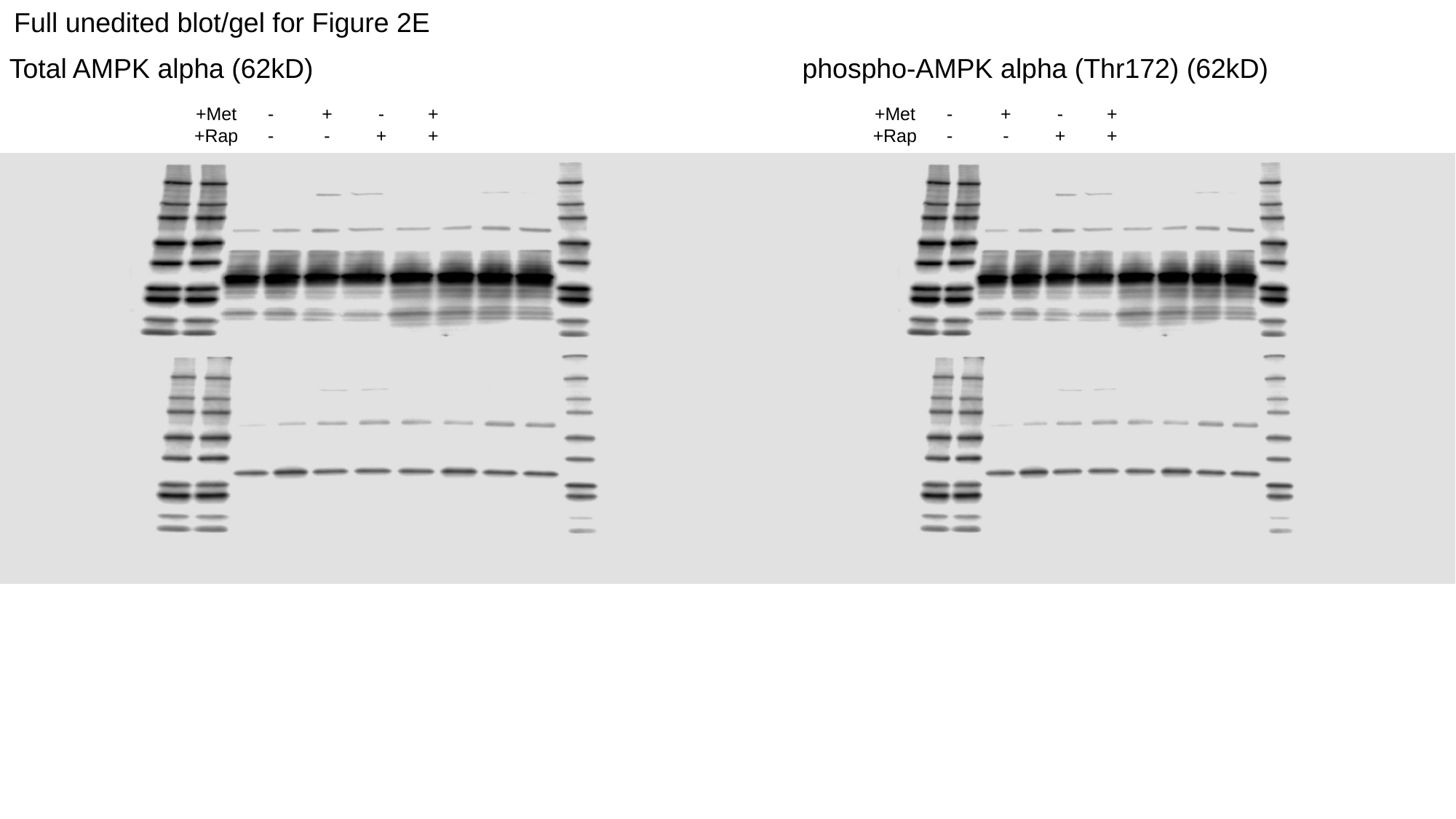

Full unedited blot/gel for Figure 2E
Total AMPK alpha (62kD)
phospho-AMPK alpha (Thr172) (62kD)
+Met
+Rap
-
-
+
-
-
+
+
+
+Met
+Rap
-
-
+
-
-
+
+
+

## Slide 2
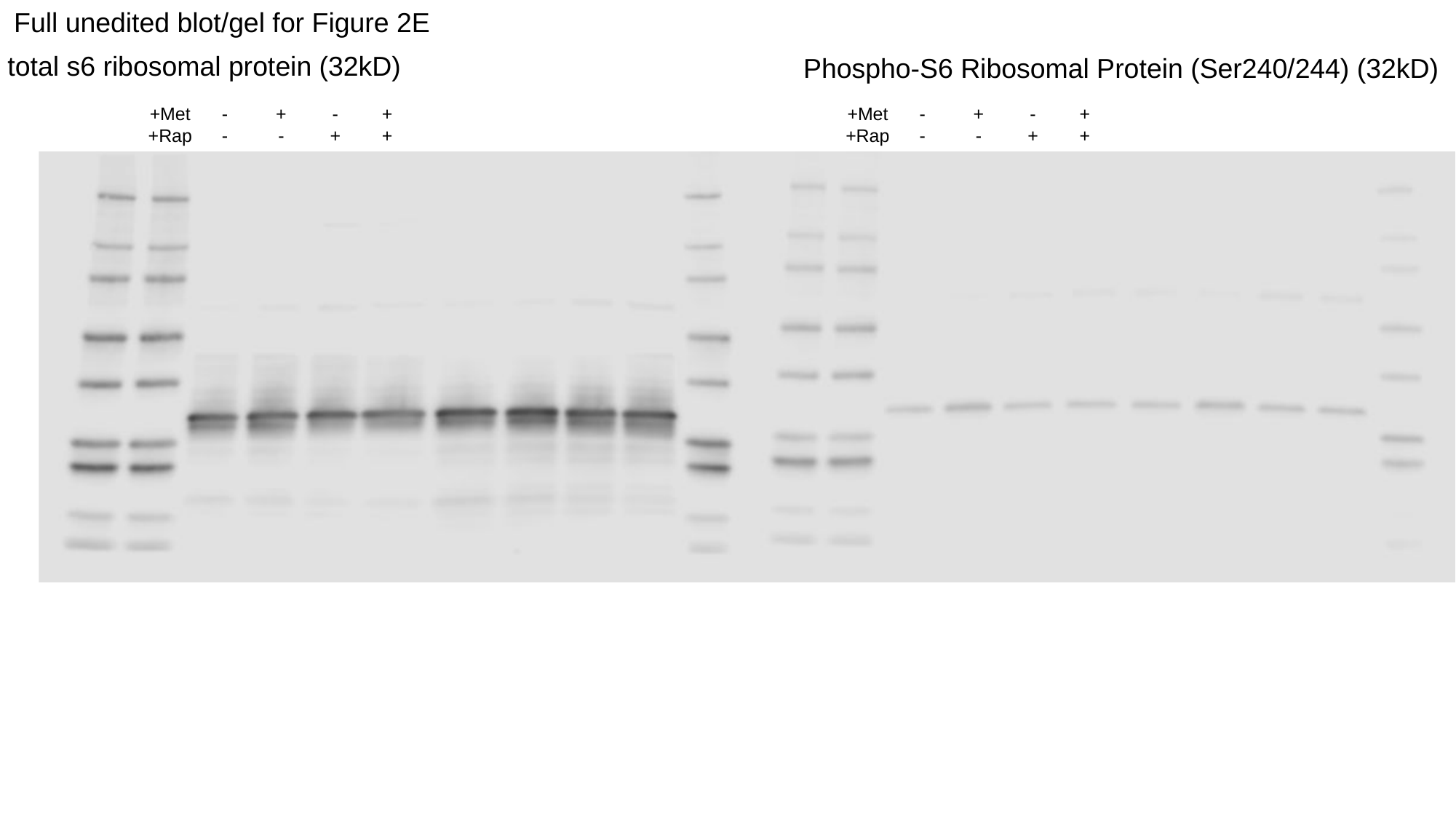

Full unedited blot/gel for Figure 2E
total s6 ribosomal protein (32kD)
Phospho-S6 Ribosomal Protein (Ser240/244) (32kD)
+Met
+Rap
-
-
+
-
-
+
+
+
+Met
+Rap
-
-
+
-
-
+
+
+

## Slide 3
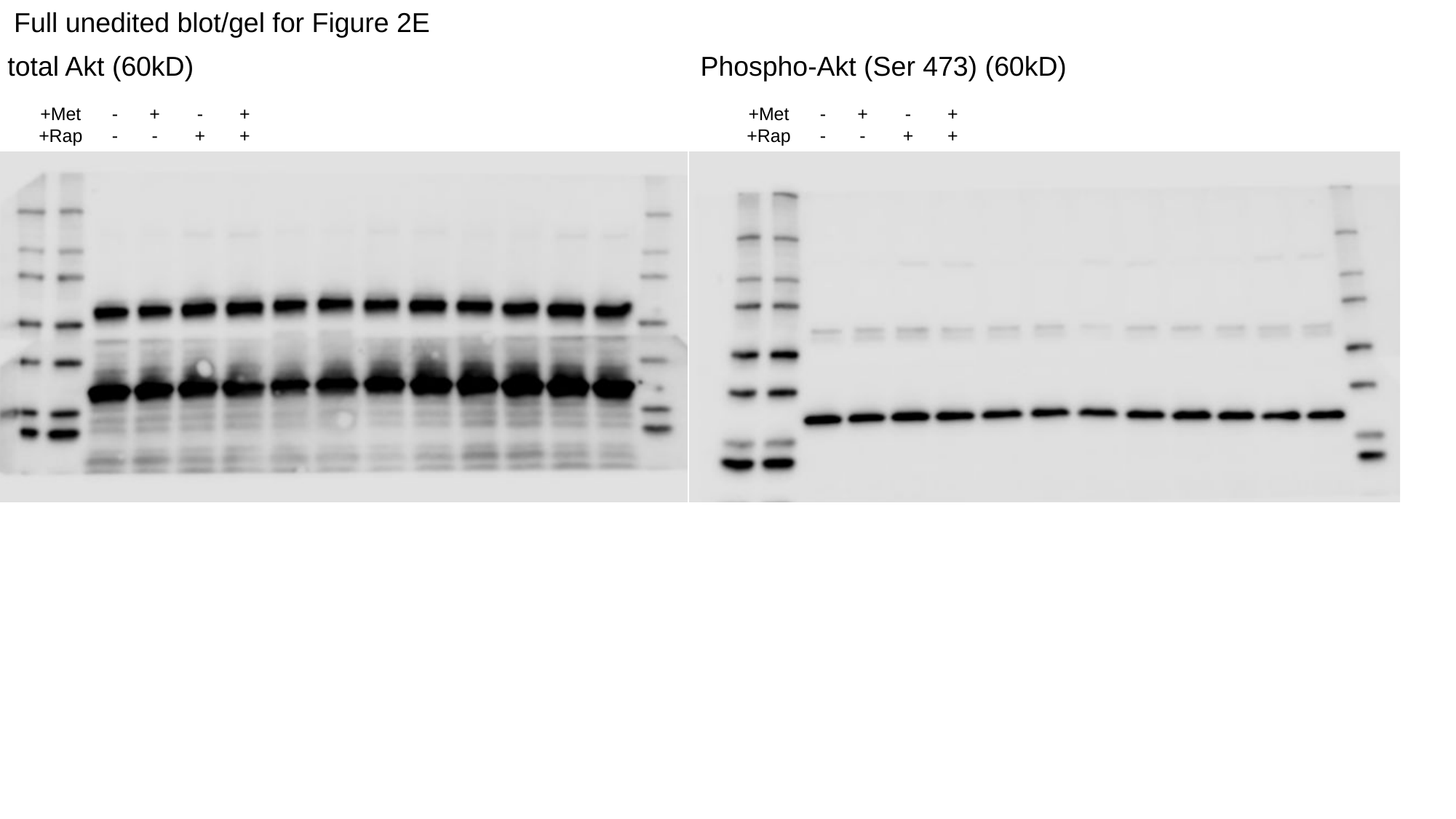

Full unedited blot/gel for Figure 2E
total Akt (60kD)
Phospho-Akt (Ser 473) (60kD)
+Met
+Rap
-
-
+
-
-
+
+
+
+Met
+Rap
-
-
+
-
-
+
+
+

## Slide 4
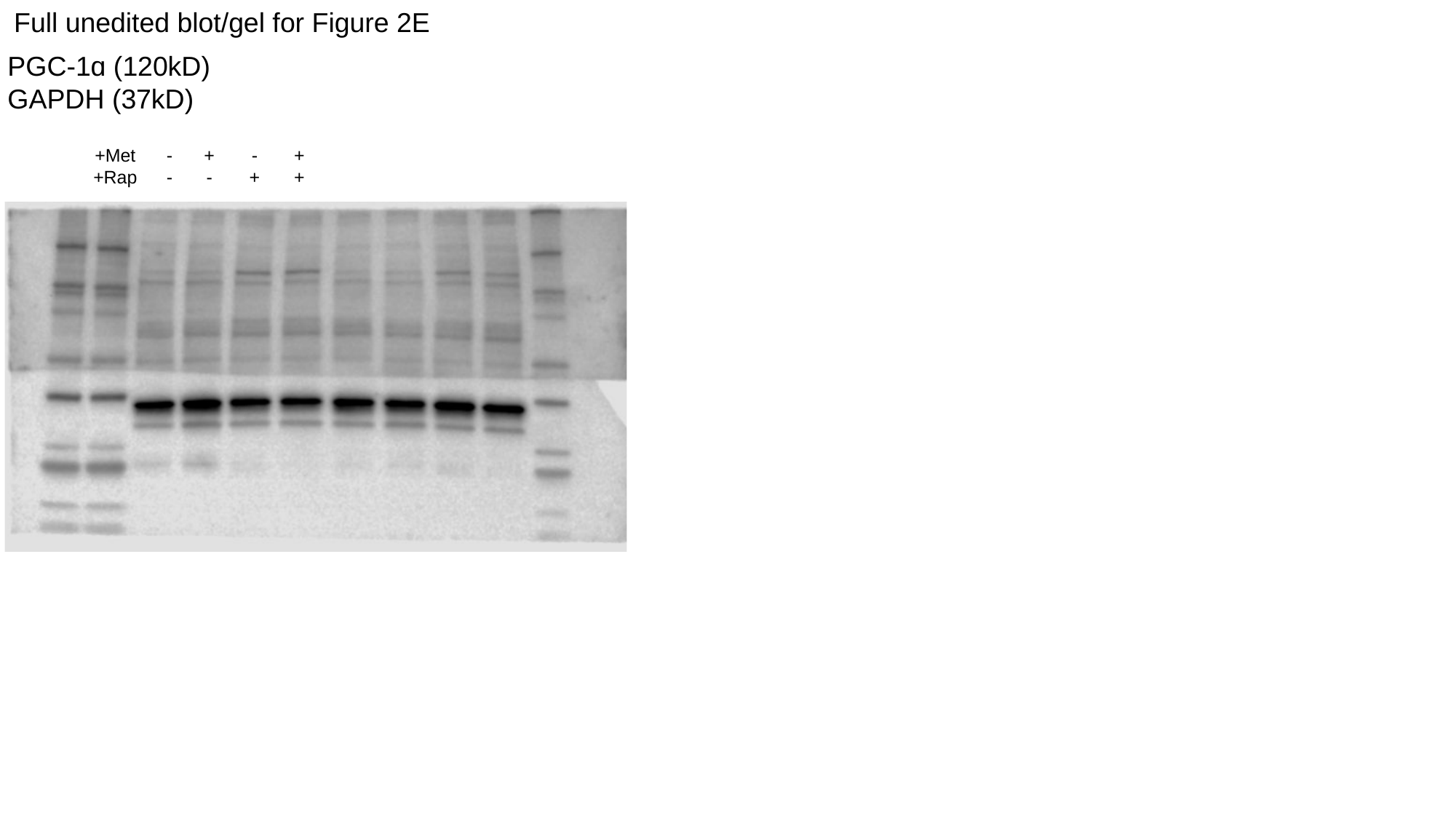

Full unedited blot/gel for Figure 2E
PGC-1ɑ (120kD)
GAPDH (37kD)
+Met
+Rap
-
-
+
-
-
+
+
+
